# Modulation of the Antimelanoma Activity Imparted to Artemisinin Hybrids by the Monoterpene Counterpart

**DOI:** 10.3390/molecules29143421

**Published:** 2024-07-21

**Authors:** Elisa De Marchi, Silvia Filippi, Silvia Cesarini, Beatrice Di Maio, Bruno Mattia Bizzarri, Raffaele Saladino, Lorenzo Botta

**Affiliations:** Department of Biological and Ecological Sciences, University of Viterbo, Via S.C. De Lellis s.n.c., 01100 Viterbo, Italy; elisa.demarchi@unitus.it (E.D.M.); silvia.filippi@unitus.it (S.F.); c.cesarinisilvia@gmail.com (S.C.); beatrice.dimaio@studenti.unitus.it (B.D.M.); bm.bizzarri@unitus.it (B.M.B.); saladino@unitus.it (R.S.)

**Keywords:** hybrids, artesunic acid, dihydroartemisinin, monoterpenes, antimelanoma activity

## Abstract

Molecular hybridization is a widely used strategy in drug discovery and development processes that consists of the combination of two bioactive compounds toward a novel entity. In the current study, two libraries of hybrid derivatives coming from the linkage of sesquiterpene counterparts dihydroartemisinin and artesunic acid, with a series of monoterpenes, were synthesized and evaluated by cell viability assay on primary and metastatic melanoma cell lines. Almost all the obtained compounds showed micromolar antimelanoma activity and selectivity toward the metastatic form of this cancer. Four hybrid derivatives containing perillyl alcohol, citronellol, and nerol as monoterpene counterpart emerged as the best compounds of the series, with nerol being active in combination with both sesquiterpenes, dihydroartemisinin and artesunic acid. Preliminary studies on the mechanism of action have shown the dependence of the pharmacological activity of newly synthesized hybrids on the formation of carbon- and oxygen-centered radical species. This study demonstrated the positive modulation of the pharmacodynamic effect of artemisinin semisynthetic derivatives dihydroartemisinin and artesunic acid due to the hybridization with monoterpene counterparts.

## 1. Introduction

**Figure 1:**
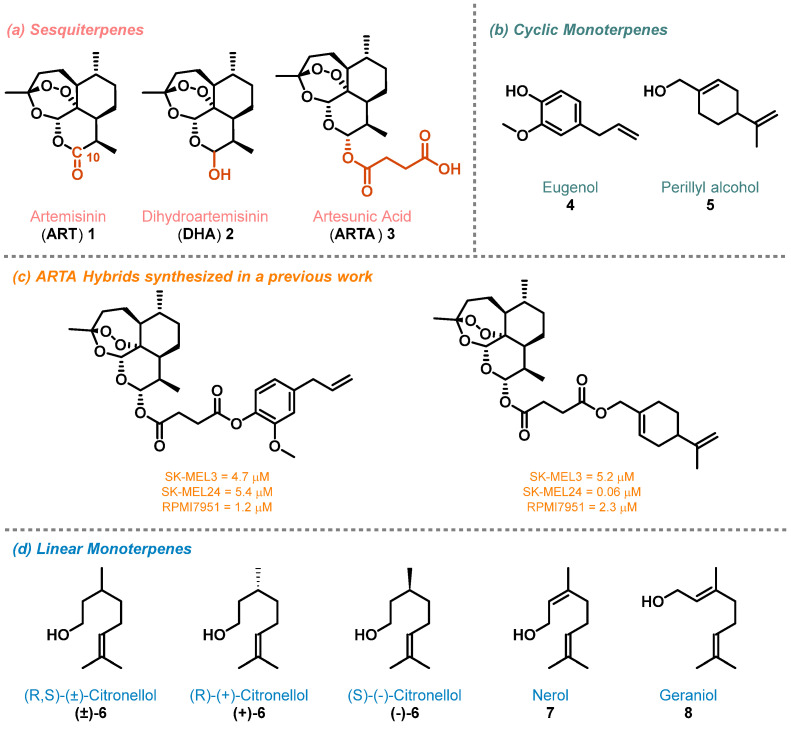
Structures of sesquiterpenes **1**–**3** (Panel **a**), cyclic monoterpenes **4**–**5** (Panel **b**), ARTA hybrid derivatives obtained in a previous study (Panel **c**) [22], and linear monoterpene **6**–**8** (Panel **d**).

Several medicinal plants, such as those of the *genus Artemisia*, have been globally employed in traditional medicine to treat various diseases, ranging from minor fevers to malaria [1]. The history of the isolation of the sesquiterpene lactone artemisinin (ART, **1**; Figure 1a) from *Artemisia annua* L. and its antimalarial, antimicrobial, and antiviral properties are reviewed and discussed in detail [2,3,4]. Interestingly, ART has also shown wide anticancer activities associated with an untargeted biological mechanism that requires initial breakage of the endoperoxide pharmacophore catalyzed by iron (Fe^2+^), with subsequent generation of highly reactive carbon- and oxygen-centered radical species [5,6,7]. This mode of action gives ART excellent selectivity against cancer cell lines which have a higher concentration of Fe^2+^, with respect to normal cells, and a diminished expression of antioxidant enzymes able to scavenge radicals [8]. Unfortunately, ART has some limitations, such as a short pharmacological half-life [9], poor solubility [10], and reduced bioavailability, that limit its use in cancer treatment [11]. For this reason, two semisynthetic derivatives of ART, C-10 lactol dihydroartemisinin (DHA, **2**) and C-10 hemisuccinate artesunic acid (ARTA, **3**), were synthesized with the aim to ameliorate the pharmacokinetic properties of the parent compound [11] (Figure 1a). It is noteworthy that **2** and **3** maintain the pharmacodynamic properties of **1** being effective on malaria [11]; viruses [4]; and some types of cancers, such as melanoma [12].

Melanoma is a neoplasm that is, in most cases, localized, and is usually treated by surgery [13]. The metastatic forms of this cancer, instead, require the use of conventional drugs like temozolomide (IC_50_ < 50 μM) [14], dacarbazine (IC_50_ between 0.12 and 1.2 μM), [15] and paclitaxel, or antibodies, and specific inhibitors of the BRAF-kinase, such as vemurafenib (IC_50_ between 0.03 and 7.2 μM) [16,17]. Chemotherapy, due to the high rate of mutation of this cancer, often becomes ineffective for the emergence of drug-resistance mechanisms [18]. A possible way to overcome resistance phenomena is the use of the molecular hybridization. The latter is a medicinal chemistry strategy based on the combination of two or more biologically relevant products, often of natural origin, to produce a new molecule, namely a hybrid derivative, with improved pharmacological activity and pharmacokinetic profile compared to parent compounds [19,20,21].

Recently, our group reported the synthesis and the antimelanoma evaluation of hybrids and dimers of DHA and ARTA with phytochemical products [22]. In particular, starting from cyclic monoterpenes found in the extract of *Artemisia annua*, eugenol **4** and perillyl alcohol **5** (Figure 1b) [23,24], were obtained the corresponding ARTA hybrids that showed low micromolar activity on three metastatic melanoma cell lines: SK-MEL3, SK-MEL24, and RPMI7951 (Figure 1c). Moved by the interesting results obtained, in the present study, we decided to synthesize additional sesquiterpene/monoterpene hybrids with the aim to identify potential antimelanoma agents. In detail, a series of monoterpenes, including cyclic **4** and **5**; linear citronellol in its racemic **(±)-6**, enantiopure **(+)-6,** and **(-)-6** forms; and nerol **7** and its geometric isomer geraniol **8** (Figure 1b,d), were chosen as counterparts for sesquiterpenes **2** and **3**. A new library of hybrids bearing a cleavable ester linker was obtained by connecting ARTA **3** and monoterpenes **4**–**8**. A second library of compounds characterized by a more stable ether linker was produced using DHA **2** as sesquiterpene’s counterpart. All the obtained compounds were assayed on primary and metastatic melanoma cell lines derived from the same patient by [3-(4,5-dimethylthiazol-2-yl)-2,5-diphenyl-2H-tetrazolium bromide] (MTT) assays. Finally, preliminary studies on the mechanism of action and the dependence of the pharmacological effect on the formation of carbon- and oxygen-centered radicals were conducted by evaluating cell viability in the presence of an iron-sequestering reagent. Cell viability tests were also conducted on the analogues of the more potent DHA hybrids lacking the endoperoxide moiety with the scope to demonstrate the importance of this pharmacophore for the generation of radical species in the cell.

## 2. Results and Discussion

**Figure 2:**
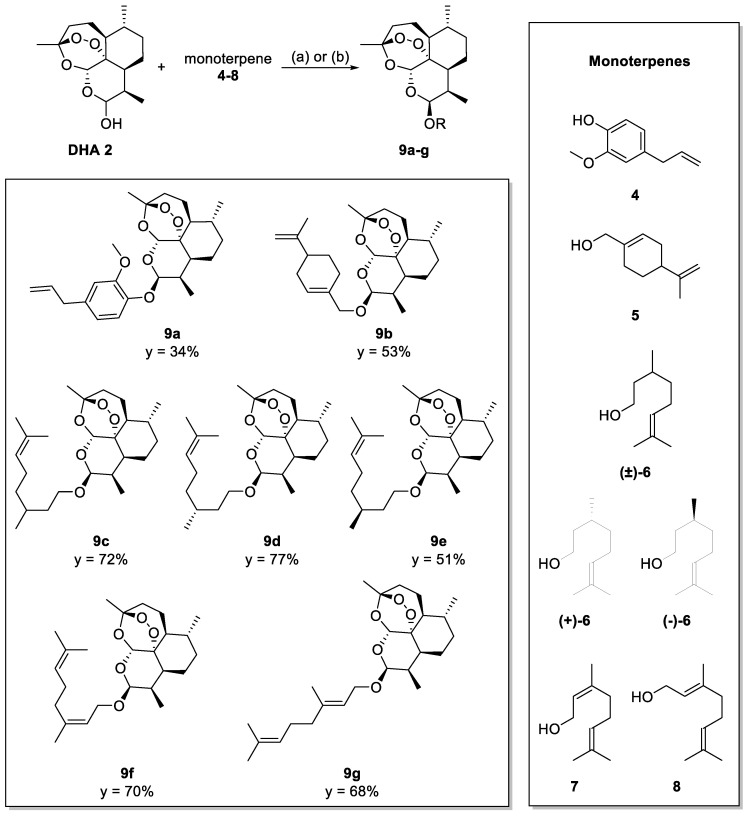
Synthesis of the DHA–hybrids **9a**–**g**. Reaction conditions: (a) boron trifluoride diethyl etherate (BF_3_ ⋅ Et_2_O) and diethyl ether (Et_2_O), 0 °C, 3 h; (b) DIAD and PPh_3_, 0 °C to r.t, 24 h. Y = yield after chromatographic purification.

Hybrid derivatives **9a**–**g** characterized by an ether bond between sesquiterpene and monoterpene counterparts were synthesized by two different strategies, as depicted in Figure 2. In the case of aromatic eugenol **4**, DHA **2** was subjected to Mitsunobu reaction in the presence of diisopropylazodicarboxylate (DIAD) and triphenylphosphine (PPh_3_) to obtain compound **9a**. For aliphatic monoterpenes **5**–**8**, instead, a Lewis acid-catalyzed coupling was used to afford products **9b**–**g** (Figure 2). Both the procedures employed gave exclusively the C-10 β-epimer, as clearly assigned by ^1^H NMR on the basis of the coupling constant between adjacent H-10 and H-9 protons [*J*_(H9, H10)_ = 3.2–3.3 Hz] when in cis-configuration [25].

**Figure 3:**
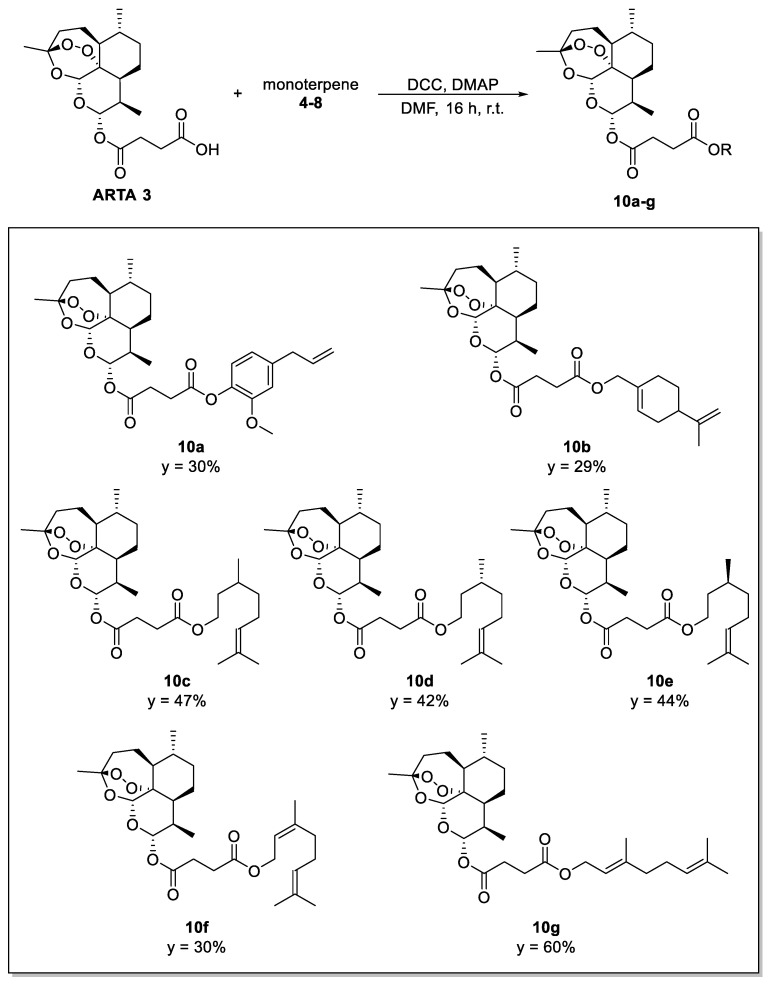
Synthesis of the ARTA–hybrids **10a**–**g**. Reaction conditions: DCC, DMAP, dimethylformamide (DMF), r.t., 16 h. Y = yield after chromatographic purification.

ARTA hybrid compounds **10a**–**g** (Figure 3), characterized by a cleavable linker, were synthesized by Steglich esterification between sesquiterpene **3** (1.0 mmol) and the appropriate monoterpene **4**–**8** (0.7 mmol), using *N*,*N*′-dicyclohexylcarbodiimide (DCC; 1.2 mmol) and 4-dimethylaminopyridine (DMAP; 0.17 mmol) as coupling agents (Figure 3). Note that even eugenol and perillyl alcohol hybrids obtained in the previous study [22] abovementioned were re-synthesized with this procedure.

**Figure 4:**
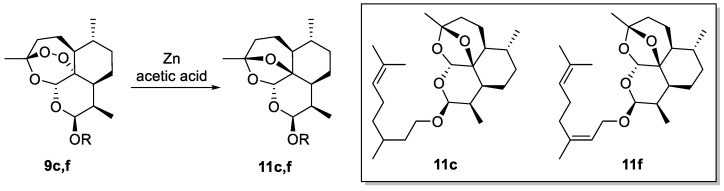
Synthesis of 2-deoxydihydroartemisinin derivatives **11c**,**f** starting from DHA hybrids **9c**,**f**.

In order to demonstrate a potential correlation between the biological activity and the formation of carbon- and oxygen-centered radicals, the pharmacophoric endoperoxide bridge of the most effective DHA hybrid derivatives **9c**,**f** was reduced to a cyclic ether function. In detail, compounds **9c** and **f** were converted into 2-deoxydihydroartemisinin **11c**,**f** via a reaction with zinc (Zn) and acetic acid (CH_3_COOH) at room temperature (Figure 4) [26].

Next, we evaluated the anticancer activity of hybrid derivatives **9a**–**g** and **10a**–**g** via a cell survival MTT assay on both primary melanoma cell line WM115 and metastatic melanoma cell line WM266, using normal fibroblasts, C3PV, as reference. DHA **2**, ARTA **3**, monoterpenes **4**–**8**, and the well-known anticancer drug, paclitaxel (Taxol; PTX), were used as standards. The selectivity of tested compounds toward cancer cells vs. normal ones was disclosed via the tumor selectivity index (TSI), calculated as the ratio between the half-maximal inhibitory concentration (IC_50_) value on C3PV and the IC_50_ values on WM115 and WM266, respectively (Table 1).

As depicted in Table 1, sesquiterpenes **2** and **3** showed micromolar activity on melanoma cell lines accompanied by cytotoxicity of the same order of magnitude, resulting in TSI values between 0.4 and 1.3 (Table 1, entries 1 and 2). PTX, on the other hand, was demonstrated to be active on cancer cell lines, particularly the metastatic one, and poorly cytotoxic on C3PV, giving TSI values of 34.3 and 87.7 with respect to WM115 and WM266 (Table 1, entry 24).

The assays conducted on monoterpenes **4**–**8** showed greater antitumor activity on the metastatic WM266 cell line than on the primary WM115 line, a trend that also occurred in the hybrid derivatives. Specifically, perillyl alcohol **5** highlighted a good activity on tumor lines, along with low cytotoxicity on fibroblasts (Table 1, entry 4). Also noteworthy are the TSI values of citronellol **(±)-6** and nerol **7** on WM266 of 10 and 7.6, respectively (Table 1, entries 5 and 8).

As mentioned above, in most cases, the hybrids resulted in being more effective on metastatic cancer cells than on primary ones regardless of their sesquiterpene counterparts. Among the compounds synthesized, the hybrid between DHA and racemic citronellol **9c** and that between ARTA and perillyl alcohol **10b** are the most potent (Table 1, entries 12 and 18). The former, due to the very low cytotoxicity on healthy fibroblast has TSI values of 173.4 and 260.1 toward WM115 and WM266. Surprisingly, compounds bearing the enantiopure forms of citronellol **9d** and **9e** have good data of antimelanoma activity and selectivity toward cancer cells but not of the same order of magnitude as **9c** (Table 1, entries 13 and 14 vs. entry 12). The TSI values of compound **10b** are 676.7 and 1015.0 by virtue of its nanomolar anticancer activity accompanied, however, by discrete cytotoxicity on C3PV. Note that **10b** is two orders of magnitude more potent against the WM115 cell line and one order of magnitude against WM266 than PTX (Table 2, entry 24 vs. entry 18), and two orders on both the lines with respect to the parent compound ARTA (Table 2, entry 2 vs. entry 18). The hybrids of nerol with both DHA (**9f**) and ARTA (**10f**) showed good activity, especially toward the metastatic WM266 line (Table 1, entries 15 and 22).

The compounds, on the other hand, endowed with lowest-activity data and consequently the worst TSI values were hybrids **9g** and **10g**, coming from the combination of **2** and **3** with geraniol **8** (Table 1, entries 16 and 23). The latter, which differs from citronellol **(±)-6** by the presence of an additional double bond and is a geometric isomer of nerol **7**, gave the least effective derivatives independently of its sesquiterpene counterpart.

Finally, cyclic ether analogues of the more potent DHA hybrids, lacking the endoperoxide pharmacophoric group, showed decreased activity on the tested WM266 line. 2-deoxyartemisinin **11c** and **f**, in fact, have a 25- and 40-fold lower antimelanoma effect compared to the corresponding compounds **9c** and **f**, respectively (Table 2, entries 25 and 26 vs. 12 and 15).

Based on cellular results, hybrids **9c**, **9f**, **10b**, and **10f** were selected for further studies. Their stability was evaluated by NMR analyses after heating them at 45 and 100 °C for 8 and 2 h, respectively (Supplementary Materials S1). After this time, ^1^H NMR of all the four compounds showed less than 5% decomposition, confirming their stability irrespectively from the ester or ether linker.

In order to prove the importance of the hybridization to ameliorate the pharmacodynamic properties of the newly synthesized derivatives, co-administration studies on the WM266 cell line were conducted. In this assay, equimolar (1:1 mmol/mmol) portions of individual components of **9c**, **9f**, **10b**, and **10f** were co-injected in the cell culture medium during MTT tests. As shown in Table 2, in all the cases, the potency of the combination of the unfastened parent compounds is lower compared to the corresponding hybrid derivatives. The more pronounced decrease in antimelanoma activity was registered for ARTA combinations, where ARTA+nerol showed a decrease by one order of magnitude, and ARTA+perillyl alcohol by two orders (Table 2, entries 3 and 4).

To obtain information about the mode of action of compounds **9c**, **9f**, **10b**, and **10f**, we repeated the cell viability assays on the metastatic cancer line WM266 in the presence of the Fe^2+^ chelating agent deferoxamine (DFO). As mentioned above, Fe^2+^ ions trigger endoperoxide bridge opening of the sesquiterpene counterpart by Fenton-like mechanisms, leading to the formation of carbon- and oxygen-centered radicals. Usually, this in cell formation of single-electron reactive species seems to be responsible for the biological activity of ART and its semisynthetic and hybrid derivatives. Sequestration of Fe^2^ by DFO generally reduces the efficacy of the compounds studied. As expected, the addition of the chelating agent led to a marked decrease in potency for all the four compounds, confirming a correlation of the pharmacologic effect of these derivatives with the formation of radical species during the 48 h duration of the assay (Table 3).

## 3. Materials and Methods

### 3.1. Chemistry—General Part

Commercially available reagents were used without further purification. Chromatographic separations were performed on Merck silica gel 60 (230–400 mesh). R*_f_* values refer to TLC carried out on 0.25 mm silica gel plates (F254) with the same eluent indicated for column chromatography. The detection occurred via fluorescence quenching or development in a molybdato phosphate solution (10% in EtOH). All products were dried in high vacuum (10-3 mbar) before characterization. ^1^H NMR and ^13^C NMR spectra were measured on a Bruker Avance DRX400 (400 MHz/100 MHz) spectrometer. Chemical shifts for protons are reported in parts per million (δ scale) and internally referenced to CDCl_3_ signal at δ 7.28 and 77.0 for ^1^H and ^13^C, respectively. Coupling constants (*J*) are reported in Hz. Multiplicities are reported in the conventional form: s = singlet, d = doublet, t = triplet, dd = doublet of doublets, q = quartet, m = multiplet, and br s = broad singlet. Mass spectra of compounds were recorded using a Vanquish HPLC system coupled to an ISQ EC single-quadrupole mass spectrometer (Thermo Fisher Sci., Waltham, MA, USA). Fourier-transform infrared spectral analysis (FTIR) was carried out using Shimadzu spirit QATR-S instrument (compounds **9a**–**g**) and an Agilent Cary 630 FT-IR spectrometer (UATR unit cell) on an ATR mode (compounds **10a**–**g**). Dihydroartemisinin and artesunic acid were obtained from Lachifarma s.r.l. (Zollino (LE), Italy).

### 3.2. Chemistry—Experimental Procedures and Compound Characterization

#### 3.2.1. Procedure for the Synthesis of Hybrids **9a**

DHA **2** (150 mg, 1.0 eq., 0.53 mmol) and eugenol **4** (1.0 eq., 0.53 mmol) were dissolved in toluene dry (6.0 mL) and DMF dry (0.47 mL) under inert atmosphere at 0 °C. To the obtained mixture were added DIAD (1.0 eq., 0.53 mmol) and PPh_3_ (1.0 eq., 0.53 mmol), and the reaction was stirred under magnetic agitation at room temperature for 24 h. After this time, the reaction was evaporated under reduced pressure, the residue was diluted in AcOEt (20 mL), and the organic layer was washed with LiCl 3% (3 × 20.0 mL) and brine (1 × 20.0 mL). The organic layer was collected, dried over sodium sulfate (Na_2_SO_4_), filtered, and evaporated under reduced pressure. The residue was purified by column chromatography, obtaining the desired product in a 34% yield (10β isomer; R*_f_* = 0.27, PE/AcOEt 10:3, molybdato phosphate stain). ^1^H NMR (CDCl_3_, 400 MHz): *δ* = 7.09 (d, 1H, *J* = 7.48 Hz), 6.74–6.72 (m, 2H), 6.02–5.91 (m, 1H), 5.68 (s, 1H), 5.46 (d, 1H, *J* = 3 Hz), 5.12–5.06 (m, 2H), 3.82 (s, 3H), 3.34 (d, 2H, *J =* 7.48 Hz), 2.82–2.78 (m, 1H), 2.44–2.36 (m, 1H), 2.22–2.11 (m, 1H), 2.07–2.03 (m, 1H), 1.94–1.89 (m, 2H), 1.75–1.71 (m, 1H), 1.61-1.43 (m, 3H), 1.43 (s, 3H), 1.36–1.23 (m, 2H), 1.10 (d, 3H, *J* = 7.2 Hz), 1.00 (d, 3H, *J* = 6.4 Hz) ppm. ^13^C NMR (CDCl_3_, 100 MHz): *δ =* 150.97, 145.03, 137.63, 135.01, 121.01, 118.92, 115.56, 113.05, 104.06, 102.21, 88.42, 81.14, 56.02, 52.71, 44.57, 39.92, 37.53, 36.48, 34.86, 31.18, 26.10, 24.72, 24.34, 20.38, 13.09 ppm. MS (ESI) *m*/*z* calcd. for [C_25_H_34_O_6_+Na]^+^ = 453.2248; found = 453.2. IR (film) νmax 2923.54, 2871.90, 1509.11, 1448.86, 1375.70, 1263.81, 1093.10, 1032.85 cm^−1^.

#### 3.2.2. General Procedure for the Synthesis of Hybrids **9b–g**

To a solution of DHA **2** (150 mg, 1.0 eq., 0.53 mmol) and the selected monoterpene **5**–**8** (1.0 eq., 0.53 mmol) in anhydrous Et_2_O (18.0 mL) at 0 °C was added BF_3_ ⋅ Et_2_O (1 eq., 0.53 mmol), and the mixture was stirred at 0 °C under a N_2_ atmosphere. After 3 h, the reaction was stopped by adding saturated aqueous NaHCO_3_ solution (10.0 mL), the organic and aqueous layers were separated, and the aqueous one was extracted with Et_2_O (3 × 20.0 mL). The combined organic layers were dried over sodium sulfate (Na_2_SO_4_), filtered, and evaporated under reduced pressure. The residue was purified by column chromatography, obtaining the desired product.

Hybrid **9b**

Yield: 53% (10 β isomer). R*_f_* = 0.78 (PE/Et_2_O 1:1, molybdato phosphate stain). ^1^H NMR (CDCl_3_, 400 MHz): *δ* = 5.71 (br s, 1H), 5.44 (s, 1H), 4.83 (d, 1H, *J* = 3.3 Hz), 4.73 (s, 2H), 4.18 (d, 1H, *J* = 12.1 Hz), 3.90 (d, 1H, *J* = 12.1 Hz), 2.68–2.64 (m, 1H), 2.43–2.35 (m, 2H), 2.19–2.14 (m, 2H), 2.08–2.01 (m, 3H), 1.98–1.78 (m, 4H), 1.76 (s, 3H), 1.69–1.44 (m, 4H), 1.46 (s, 3H), 1.39–1.33 (m, 1H), 1.30–1.22 (m, 2H), 0.95 (dd, 6H, *J* = 6.3, 12.4 Hz) ppm. ^13^C NMR (CDCl_3_, 100 MHz): *δ =* 149.91, 134.31, 123.70, 108.61, 104.07, 100.73, 87.97, 81.22, 71.84, 52.62, 44.48, 41.15, 37.43, 36.47, 34.68, 30.94, 30.49, 27.48, 26.38, 24.71, 24.52, 20.79, 20.39, 13.11 ppm. MS (ESI) *m*/*z* calcd. for [C_25_H_38_O_5_ + Na]^+^ = 441.2611; found = 441.2. IR (film) νmax 2923.54, 2851.82, 1685.56, 1453.17, 1372.83, 1199.26, 1111.75, 991.25 cm^−1^.

Hybrid **9c**

Yield: 72% (10β isomer). R*_f_* = 0.80 (PE/Et_2_O 2:1, molybdato phosphate stain). ^1^H NMR (CDCl_3_, 400 MHz): *δ* = 5.41 (s, 1H), 5.11 (t, 1H, *J* = 7.0 Hz), 4.79 (d, 1H, *J* = 3.3 Hz), 3.94–3.86 (m, 1H), 3.45–3.36 (m, 1H), 2.65–2.61 (m, 1H), 2.43–2.35 (m, 1H), 2.08–1.86 (m, 5H), 1.82–1.72 (m, 2H), 1.70 (s, 3H), 1.67–1.53 (m, 4H), 1.62 (s, 3H), 1.51–1.46 (m, 2H), 1.46 (s, 3H), 1.42–1.31 (m, 4H), 1.29–1.13 (m, 3H), 0.97 (d, 3H, *J* = 6.2 Hz), 0.93–0.89 (m, 3H) ppm. ^13^C NMR (CDCl_3_, 100 MHz): *δ =* 131.17, 124.77, 104.05, 101.96, 87.95, 81.17, 66.76, 52.63, 44.52, 37.49, 37.14, 36.67, 36.48, 34.70, 30.93, 29.60, 26.25, 25.73, 25.50, 24.70, 24.45, 20.40, 19.56, 17.65, 13.05 ppm. MS (ESI) *m*/*z* calcd. for [C_25_H_42_O_5_ + Na]^+^ = 445.2924; found = 445.3. IR (film) νmax 2923.54, 2850.38, 1685.56, 1453.17, 1372.83, 1199.26, 1110.32, 991.25 cm^−1^.

Hybrid **9d**

Yield: 77% (10β isomer). R*_f_* = 0.80 (PE/Et_2_O 2:1, molybdato phosphate stain). ^1^H NMR (CDCl_3_, 400 MHz): *δ* = 5.41 (s, 1H), 5.11 (t, 1H, *J* = 7.0 Hz), 4.79 (d, 1H, *J* = 3.3 Hz), 3.94–3.88 (m, 1H), 3.42–3.36 (m, 1H), 2.65–2.61 (m, 1H), 2.43–2.35 (m, 1H), 2.08–1.86 (m, 5H), 1.83–1.72 (m, 2H), 1.70 (s, 3H), 1.67–1.53 (m, 4H), 1.62 (s, 3H), 1.51–1.45 (m, 2H), 1.46 (s, 3H), 1.42–1.32 (m, 4H), 1.30–1.13 (m, 3H), 0.97 (d, 3H, *J* = 6.2 Hz), 0.93–0.89 (m, 3H) ppm. ^13^C NMR (CDCl_3_, 100 MHz): *δ =* 131.14, 124.79, 104.04, 102.15, 87.95, 81.18, 66.86, 52.63, 44.52, 37.48, 37.08, 36.81, 36.48, 34.70, 30.97, 29.58, 26.25, 25.72, 25.54, 24.70, 24.44, 20.39, 19.56, 17.65, 13.03 ppm. MS (ESI) *m*/*z* calcd. for [C_25_H_42_O_5_ + Na]^+^ = 445.2924; found = 445.3. IR (film) νmax 2923.54, 2851.82, 1685.56, 1453.17, 1372.83, 1199.26, 1111.75, 991.25 cm^−1^.

Hybrid **9e**

Yield: 51% (10β isomer). R*_f_* = 0.80 (PE/Et_2_O 2:1, molybdato phosphate stain). ^1^H NMR (CDCl_3_, 400 MHz): *δ* = 5.41 (s, 1H), 5.11 (t, 1H, *J* = 7.0 Hz), 4.80 (d, 1H, *J* = 3.2 Hz), 3.91–3.86 (m, 1H), 3.45–3.39 (m, 1H), 2.65–2.61 (m, 1H), 2.43–2.35 (m, 1H), 2.07–1.85 (m, 5H), 1.83–1.73 (m, 2H), 1.70 (s, 3H), 1.67–1.54 (m, 4H), 1.62 (s, 3H), 1.51–1.46 (m, 2H), 1.46 (s, 3H), 1.43–1.31 (m, 4H), 1.29–1.11 (m, 3H), 0.97 (d, 3H, *J* = 6.2 Hz), 0.92 (d, 3H, *J* = 6.8 Hz) ppm. ^13^C NMR (CDCl_3_, 100 MHz): *δ =* 131.17, 124.77, 104.05, 101.96, 87.95, 81.17, 66.76, 52.63, 44.52, 37.49, 37.14, 36.67, 36.48, 34.70, 30.93, 29.60, 26.25, 25.73, 25.50, 24.70, 24.45, 20.40, 19.56, 17.65, 13.05 ppm. MS (ESI) *m*/*z* calcd. for [C_25_H_42_O_5_ + Na]^+^ = 445.2924; found = 445.3. IR (film) νmax 2923.54, 2850.38, 1685.56, 1453.17, 1372.83, 1199.26, 1110.32, 991.25 cm^−1^.

Hybrid **9f**

Yield: 70% (10β isomer). R*_f_* = 0.80 (PE/Et_2_O 1:1, molybdato phosphate stain). ^1^H NMR (CDCl_3_, 400 MHz): *δ* = 5.43 (s, 1H), 5.31 (t, 1H, *J* = 7.0 Hz), 5.11 (br s, 1H), 4.83 (d, 1H, *J* = 3.3 Hz), 4.31 (dd, 1H, *J* = 6.2, 5.9 Hz), 4.01 (dd, 1H, *J* = 7.1, 5 Hz), 2.66–2.62 (m, 1H), 2.47–2.35 (m, 1H), 2.14–2.03 (m, 5H), 1.96–1.79 (m, 3H), 1.76 (s, 3H), 1.70 (s, 3H), 1.62 (s, 3H), 1.76-1.62 (m, 2H), 1.57–1.48 (m, 1H), 1.45 (s, 3H), 1.42– 1.07 (m, 3H), 0.97 (d, 3H, *J* = 5.9,Hz) 0.92 (d, 3H, *J* = 7.3 Hz) ppm. ^13^C NMR (CDCl_3_, 100 MHz): *δ =* 139.80, 131.83, 123.91, 121.01, 104.02, 101.30, 87.97, 81.19, 64.64, 52.66, 44.57, 39.53, 37.50, 36.51, 34.70, 30.91, 26.72, 26.20, 25.65, 24.72, 24.51, 20.35, 17.67, 16.60, 13.03 ppm. MS (ESI) *m*/*z* calcd. for [C_25_H_40_O_5_ + Na]^+^ = 443.2768; found = 443.3. IR (film) νmax 2923.54, 2851.82, 1685.56, 1453.17, 1372.83, 1199.26, 1111.75, 991.25 cm^−1^.

Hybrid **9g**

Yield: 68% (10β isomer). R*_f_* = 0.81 (PE/Et_2_O 1:1, molybdato phosphate stain). ^1^H NMR (CDCl_3_, 400 MHz): *δ* = 5.44 (s, 1H), 5.30 (t, 1H, *J* = 7.0 Hz), 5.11 (t, 1H, *J* = 6.6 Hz), 4.84 (d, 1H, *J* = 3.3 Hz), 4.30 (dd, 1H, *J* = 6.0, 6.2 Hz), 4.06 (dd, 1H, *J* = 7.1, 5.2 Hz), 2.66–2.62 (m, 1H), 2.43–2.35 (m, 1H), 2.15–2.03 (m, 5H), 1.93–1.73 (m, 3H), 1.70 (s, 3H), 1.67 (s, 3H), 1.66–1.56 (m, 1H), 1.63 (s, 3H), 1.54–1.48 (m, 1H), 1.47 (s, 3H), 1.39-1.33 (m, 1H), 1.29–1.22 (m, 2H), 0.97 (d, 3H, *J* = 6.3 Hz), 0.92 (d, 3H, *J* = 7.3 Hz) ppm. ^13^C NMR (CDCl_3_, 100 MHz): *δ =* 139.71, 131.61, 124.01, 121.00, 104.03, 101.02, 87.98, 81.25, 64.65, 52.62, 44.53, 39.53, 37.43, 36.48, 34.68, 30.92, 26.43, 26.24, 25.69, 24.72, 24.50, 20.38, 17.70, 16.57, 13.06 ppm. MS (ESI) *m*/*z* calcd. for [C_25_H_40_O_5_ + Na]^+^ = 443.2768; found = 443.3. IR (film) νmax 2923.54, 2851.82, 1685.56, 1453.17, 1372.83, 1199.26, 1111.75, 991.25 cm^−1^.

#### 3.2.3. General Procedure for the Synthesis of Hybrids **10a**–**g**

To a solution of ARTA (192 mg, 1.0 eq., 0.5 mmol) in dry DMF (5.0 mL), DCC (1.2 eq., 0.6 mmol) and DMAP (0.34 eq., 0.17 mmol) were added, and the mixture was stirred at room temperature for 40 min. After this time, the opportune monoterpene (0.7 eq., 0.35 mmol) was added, and the reaction mixture was slowly stirred overnight (16 h) under inert atmosphere. The reaction was stopped by filtration through a thin layer of Celite^®^, and the filter cake was diluted in CH_2_Cl_2_ (15 mL) and washed with HCl 1M (2 × 7 mL) and brine (7 mL). The organic layer was collected, dried over sodium sulfate (Na_2_SO_4_), filtered, and evaporated under reduced pressure. The crude product was purified by column chromatography obtaining desired product.

Hybrid **10a**

Yield: 30%. R*_f_* = 0.27 (PE/AcOEt 1:0.7, molybdato phosphate stain). ^1^H NMR (CDCl_3_, 400 MHz): *δ* = 6.98 (d, 1H, *J* = 8.0 Hz), 6.86 (d, 1H, *J* = 8.5 Hz), 6.79–6.76 (m, 1H), 6.02–5.92 (m, 2H), 5.84 (d, 1H, *J* = 9.9 Hz), 5.47 (s, 1H), 5.13 (d, 1H, *J* = 9.8 Hz), 5.09 (s, 1H), 3.82 (s, 3H), 3.34 (d, 2H, *J* = 4.1 Hz), 3.02–2.84 (m, 3H), 2.63–2.57 (m, 1H), 2.44-2.35 (td, 1H, *J* = 3.9, 10.5 Hz), 2.07–1.49 (m, 6H), 1.45 (s, 3H), 1.42–1.28 (m, 4H), 0.99 (d, 3H, *J* = 5.9 Hz), 0.87 (d, 3H, *J* = 7.1 Hz) ppm. ^13^C NMR (CDCl3, 100 MHz): *δ* = 170.92, 170.40, 138.95, 137.81, 137.06, 122.53, 121.20, 120.68, 116.09,112.77, 104.47, 92.26, 91.54, 80.18, 55.87, 51.62, 45.29, 40.07, 37.30, 36.26, 34.13, 31.83, 29.39, 28.76, 25.95, 24.60, 22.01, 20.20, 12.05 ppm. MS (ESI) *m*/*z* calcd. for [C_29_H_38_O_9_ + Na]^+^ = 553.2408; found = 553.2. IR (film) νmax 2926.00, 2857.00, 1753.70, 1507.70, 1457.40, 1420.10, 1377.30, 1135.00, 1015.70 cm^−1^.

Hybrid **10b**

Yield: 29%. R*_f_* = 0.27 (PE/AcOEt 1:0.7, molybdato phosphate stain). ^1^H NMR (CDCl_3_, 400 MHz): *δ* = 5.82 (d, 1H, *J* = 9.8 Hz), 5.77 (br s, 1H), 5.45 (s, 1H), 4.74 (d, 2H, *J* = 7.3 Hz), 4.50 (s, 2H), 2.78–2.57 (m, 5H), 2.44–2.36 (td, 1H, *J* = 3.9, 9.9 Hz), 2.20–1.72 (m, 10H), 1.76 (s, 3H), 1.66–1.48 (m, 3H), 1.45 (s, 3H), 1.42–1.28 (m, 4H), 0.98 (d, 3H, *J* = 5.9 Hz), 0.87 (d, 3H, *J* = 7.1 Hz) ppm. ^13^C NMR (CDCl_3_, 100 MHz): *δ* = 171.97, 171.12, 149.60, 132.50, 125.94, 108.76, 104.46, 92.19, 91.52, 80.11, 68.75, 51.61, 45.29, 40.81, 37.29, 34.12, 31.82, 30.47, 29.28, 28.95, 27.31, 26.36, 25.94, 24.59, 22.01, 20.73, 20.19, 12.03 ppm. MS (ESI) *m*/*z* calcd. for [C_29_H_42_O_8_ + Na]^+^ = 541.2772; found = 541.2. IR (film) νmax 2926.00, 2875.80, 1736.90, 1451.80, 1377.30, 1155.50, 1015.70 cm^−1^.

Hybrid **10c**

Yield: 47%. R*_f_* = 0.90 (PE/Et_2_O 2:1, molybdato phosphate stain). ^1^H NMR (CDCl_3_, 400 MHz): *δ* = 5.79 (d, 1H, J =10), 5.44 (s, 1H), 5.09 (t, 1H, *J* = 6.6 Hz), 4.15–4.11 (m, 2H), 2.74–2.57 (m, 5H), 2.38–2.34 (td, 1H, *J* = 14.4, 4.0 Hz), 2.12–1.71 (m, 10H), 1.68 (s, 3H), 1.64 (s, 3H), 1.63–1.41 (m, 4H), 1.40 (s, 3H), 1.35–1.17 (m, 3H), 0.97 (d, 3H, *J* = 6 Hz), 0.89 (dd, 6H, *J* = 6.8, 12.4 Hz) ppm. ^13^C NMR (CDCl_3_, 100 MHz): *δ* = 172.12, 171.12, 124.58, 104.44, 92.17, 91.50, 80.10, 63.33, 51.61, 45.29, 37.28, 36.97, 36.25, 35.39, 34.12, 31.81, 29.50, 29.27, 28.96, 25.92, 25.67, 25.37, 24.59, 24.28, 21.99, 20.17, 19.38, 17.62, 12.01 ppm. MS (ESI) *m*/*z* calcd. for [C_29_H_46_O_8_ + Na]^+^ = 545.3085; found = 545.3. IR (film) νmax 2927.80, 2875.60, 1735.10, 1457.40, 1377.30, 1157.30, 1015.70 cm^−1^.

Hybrid **10d**

Yield: 42%. R*_f_* = 0.9 (PE/Et_2_O 2:1, molybdato phosphate stain). ^1^H NMR (CDCl_3_, 400 MHz): *δ* = 5.80 (d, 1H, *J* = 9.8 Hz), 5.44 (s, 1H), 5.09 (t, 1H, *J* = 6.9 Hz), 4.18-4.07 (m, 2H), 2.78–2.55 (m, 5H), 2.42–2.34 (td, 1H, *J* = 14.4, 4.0 Hz), 2.06–1.71 (m, 10H), 1.69 (s, 3H), 1.66–1.46 (m, 4H), 1.61 (s, 3H), 1.44 (s, 3H), 1.40–1.16 (m, 3H), 0.97 (d, 3H, *J* = 5.8 Hz), 0.89 (dd, 6H, *J* = 6.5, 12.3 Hz) ppm. ^13^C NMR (CDCl_3_, 100 MHz): *δ* = 172.20, 171.19, 124.57, 104.48, 92.17, 91.51, 80.12, 63.37, 51.57, 45.25, 37.28, 36.97, 36.22, 35.37, 34.09, 31.80, 29.47, 29.24, 28.93, 25.93, 25.71, 25.37, 24.95, 24.58, 21.99, 20.20, 19.38, 17.65, 12.03 ppm. MS (ESI) *m*/*z* calcd. for [C_29_H_46_O_8_+Na]^+^ = 545.3085; found = 545.3. IR (film) νmax 2927.80, 2875.60, 1735.10, 1457.40, 1377.30, 1157.30, 1015.70 cm^−1^.

Hybrid **10e**

Yield: 44%. R*_f_* = 0.9 (PE/Et_2_O 2:1, molybdato phosphate stain). ^1^H NMR (CDCl_3_, 400 MHz): *δ* = 5.81 (d, 1H, *J* = 12 Hz), 5.44 (s, 1H), 5.09 (t, 1H, *J* = 7.0 Hz), 4.18–4.08 (m, 2H), 2.78–2.53 (m, 5H), 2.43–2.35 (td, 1H, *J* = 14.4, 4.0 Hz), 2.06–1.72 (m, 10H), 1.69 (s, 3H), 1.65–1.47 (m, 4H), 1.62 (s, 3H), 1.44 (s, 3H), 1.41–1.16 (m, 3H), 0.98 (d, 3H, *J* = 5.9 Hz), 0.89 (dd, 6H, *J* = 6.4, 12.3 Hz) ppm.. ^13^C NMR (CDCl_3_, 100 MHz): *δ* = 172.12, 171.11, 124.57, 104.44, 92.15, 91.49, 80.09, 63.31, 51.59, 45.26, 37.27, 36.96, 36.23, 35.37, 34.10, 31.80, 29.48, 29.25, 28.94, 25.92, 25.67, 25.36, 24.67, 24.58, 21.98, 20.17, 19.38, 17.63, 12.01 ppm. MS (ESI) *m*/*z* calcd. for [C_29_H_46_O_8_+Na]^+^ = 545.3085; found = 545.3. IR (film) νmax 2927.80, 2873.80, 1735.10, 1457.40, 1375.40, 1155.50, 1012.00 cm^−1^.

Hybrid **10f**

Yield: 29%. R*_f_* = 0.78 (PE/Et_2_O 2:1, molybdato phosphate stain). ^1^H NMR (CDCl_3_, 400 MHz): *δ* = 5.81 (d, 1H, *J* =9.6), 5.44 (s, 1H), 5.36 (t, 1H, J = 6.9), 5.09 (t, 1H, *J* = 6.6 Hz), 4.61-4.59 (m, 2H), 2.76–2.58 (m, 5H), 2.43–2.35 (td, 1H, *J* = 11.1, 3.3 Hz), 2.11–2.02 (m, 5H), 1.92-1.80 (m, 1H), 1.77 (s, 3H), 1.70 (s, 3H), 1.62 (s, 3H), 1.62–1.01 (m, 8H), 1.44 (s, 3H), 0.98 (d, 3H, *J* = 5.6 Hz), 0.87 (d, 3H, *J* = 6.8 Hz) ppm. ^13^C NMR (CDCl_3_, 100 MHz): *δ* = 172.12, 171.11, 142.59, 132.13, 123.58, 119.07, 104.44, 92.17, 91.51, 80.10, 61.39, 51.62, 45.30, 37.29, 36.26, 34.13, 32.17, 31.82, 29.28, 28.97, 26.63, 25.93, 25.65, 24.59, 23.46, 22.01, 20.18, 17.63, 12.01 ppm. MS (ESI) *m*/*z* calcd. for [C_29_H_44_O_8_+Na]^+^ = 543.2928; found = 543.3. IR (film) νmax 2927.80, 2875.60, 1735.10, 1457.40, 1377.30, 1151.70, 1010.10 cm^−1^.

Hybrid **10g**

Yield: 60%. R*_f_* = 0.77 (PE/Et_2_O 2:1, molybdato phosphate stain). ^1^H NMR (CDCl_3_, 400 MHz): *δ* = 5.81 (d, 1H, *J* = 9.8 Hz), 5.45 (s, 1H), 5.35 (t, 1H, *J* = 6.6 Hz), 5.10 (t, 1H, *J* = 5.6 Hz), 4.63 (d, 2H, *J* = 6.9 Hz), 2.77–2.59 (m, 5H), 2.39–2.35 (td, 1H, *J* = 10.9, 3.2 Hz), 2.11–2.04 (m, 5H), 1.93-1.65 (m, 6H), 1.71 (s, 3H), 1.62 (s, 3H), 1.55-1.27 (m, 6H), 1.45 (s, 3H), 0.98 (d, 3H, *J* = 5.6 Hz), 0.87 (d, 3H, *J* = 6.9 Hz) ppm. ^13^C NMR (CDCl_3_, 100 MHz): *δ* = 172.08, 171.13, 142.36, 131.80, 123.76, 118.16, 104.45, 92.17, 91.51, 80.10, 61.67, 51.61, 45.29, 39.51, 37.29, 36.25, 34.12, 31.82, 29.29, 28.97, 26.31, 25.94, 25.65, 24.60, 22.00, 20.19, 17.67, 16.46, 12.01 ppm. MS (ESI) *m*/*z* calcd. for [C_29_H_44_O_8_+Na]^+^ = 543.2928; found = 543.3. IR (film) νmax 2926.00, 2875.60, 1735.10, 1449.90, 1377.30, 1151.70, 1015.70 cm^−1^.

#### 3.2.4. General Procedure for the Synthesis of 2-Deoxy-artemisinin Derivatives **11c** and **11f**

The selected hybrid **9c** or **9f** (1.0 equiv., 0.1 mmol) was dissolved in 1.8 mL of glacial acetic acid, and the solution was stirred under argon atmosphere for 30 min. Afterward, zinc (activated with HCl; 3.0 equiv., 0.3 mmol) was added, and the reaction mixture was stirred at r.t. The completion of the reaction was checked with TLC and stopped by filtration through a thin layer of Celite^®^, and the celite was washed with EtOAc (10 mL). The organic solution was washed with water (3 × 5 mL) and a saturated aqueous solution of NaHCO_3_ (3 × 5 mL, sat.), dried over sodium sulfate (Na_2_SO_4_), filtered, and evaporated under reduced pressure. The crude product was purified via preparatory plate.

2-deoxy-artemisinin derivative **11c**

Yield: 24%. R*_f_* = 0.68 (PE/Et_2_O 9:2, molybdato phosphate stain). ^1^H NMR (CDCl_3_, 400 MHz): *δ* = 5.32 (s, 1H), 5.11 (t, 1H, *J* = 6.8 Hz), 4.76 (d, 1H, *J* = 4.3 Hz), 3.95–3.82 (m, 1H), 3.43-3.33 (m, 1H), 2.49–2.39 (m, 1H), 2.12 (s, 1H), 2.06–1.95 (m, 2H), 1.87–1.72 (m, 5H), 1.70 (s, 3H), 1.70-1.53 (m, 4H), 1.62 (s, 3H), 1.54 (s, 3H), 1.41–1.15 (m, 8H), 0.94–0.89 (m, 6H). ^13^C NMR (CDCl_3_, 100 MHz): *δ =* 131.10, 124.84, 107.97, 99.93, 94.58, 83.51, 66.96, 46.88, 41.06, 37.15, 36.73, 36.60, 35.19, 34.92, 34.60, 30.60, 29.61, 25.71, 25.52, 24.58, 22.18, 19.56, 19.08, 17.63, 12.39 ppm. MS (ESI) *m*/*z* calcd. for [C_25_H_42_O_4_+Na]^+^ = 429.2975; found = 429.3.

2-deoxy-artemisinin derivative **11f**

Yield: 25%. R*_f_* = 0.67 (PE/Et_2_O 9:2, molybdato phosphate stain). ^1^H NMR (CDCl_3_, 400 MHz): *δ* = 5.35–532 (m, 2H), 5.11 (br. s, 1H), 4.83 (d, 1H, *J* = 4.5 Hz), 4.29–4.25 (m, 1H), 4.01-3.96 (m, 1H), 2.47–2.42 (m, 1H), 2.15–2.04 (m, 4H), 1.89–1.78 (m, 3H), 1.75 (s, 3H), 1.74–1.70 (m, 2H), 1.70 (s, 3H), 1.62 (s, 3H), 160–156 (m, 2H), 1.53 (s, 3H), 1.49–1.13 (m, 4H), 0.93 (d, 3H, *J* = 7.4 Hz), 0.92 (d, 3H, *J* = 6.1 Hz) ppm. ^13^C NMR (CDCl_3_, 100 MHz): *δ =* 139.78, 131.84, 123.93, 122.03, 107.89, 98.63, 94.78, 83.46, 64.64, 46.75, 41.09, 35.18, 34.87, 34.60, 32.33, 30.63, 26.72, 25.70, 25.09, 24.48, 23.51, 22.19, 19.06, 17.67, 12.28 ppm. MS (ESI) *m*/*z* calcd. for [C_25_H_40_O_4_+Na]^+^ = 427.2819; found = 427.2.

### 3.3. Biology

#### 3.3.1. Cell Culture Conditions

The primary human healthy fibroblast C3PV cell line was grown in a culture medium containing 50% Dulbecco Modified Medium (DMEM) and 50% Ham’s F10 supplemented with 10% Fetal Bovine Serum (FBS). Primary melanoma cell line (WM1115) and metastatic melanoma cell line (WM266) were grown in Eagle Minimal Essential Medium (EMEM) supplemented with 15% FBS, 1% non-essential amino acids, and 1% Na-Piruvate. To all growing media were added 1% Pen/Strep and 1% Glutamine. All cell lines were grown in a humidified incubator (95%) with 5% CO_2_.

#### 3.3.2. General Treatment Protocol and Cell Viability Assay

To study the effect of artemisinin derivatives on cell viability, MTT assay was performed. Briefly, C3PV, WM115, and WM266 cell lines were seeded in 96-well plates (3000 cells/well in 100 μL of medium) 24 h before treatment and incubated overnight to allow for cell adherence. Afterward, the medium was replaced with a fresh one containing the appropriate dose of newly synthesized compounds (doses ranging from 0.01 to 1 μM were used for 24 h). The analyses of cell viability were performed at the end of the treatment. Triplicates were made in all experiments. After 24 h of treatment with newly synthesized compounds, the culture medium was replaced with a solution containing 0.5 mg/mL of MTT. After 3 h of incubation in an incubator, the medium was removed, and a lysis solution (10% SDS, 0.6% acetic acid in DMSO) was added to dissolve the formazan crystals. Optical density measurements were performed with a microplate reader EPOCH/2 (Biotek) with 630 nm (background) and a 570 nm filters.

#### 3.3.3. Co-Administration Analyses

To compare the activities of hybrids **9c**, **9f**, **10b**, and **10f** and the combination of their unfastened individual components, co-administration analyses were performed. The WM266 cell line was seeded in 96-well plates (3000 cells/well in 100 μL medium) and incubated overnight to allow for cell adherence. After, the medium was replaced with a fresh one containing a combination of equimolar amount (1:1) of artemisinin derivative (DHA **2** or ARTA **3**) and the monoterpene counterpart (**5**, **(±)-6** and **7**). For example, 1.0 μM dose was made by a combination of 0.5 μM of ARTA or DHA and 0.5 μM of monoterpene. The combined compounds were used at the same doses mentioned above. At the end of the treatment, cell viability assays were carried out. Triplicates were made in all experiments.

#### 3.3.4. Treatment Protocol for DFO Assay

DFO was used to study the mode of action of compounds **9c**, **9f**, **10b**, and **10f** on WM266 cell line. Twenty-four hours before treatment, cells were seeded in 96-well plates and incubated overnight. Then, cells were pre-treated with 20 μM of DFO for 1 h. After this time, two washes with PBS were performed, and, subsequently, fresh medium containing different doses of the opportune compound was added for 48h. The analyses of cell viability were performed at the end of treatment, and triplicates were made in all experiments.

#### 3.3.5. Statistical Analysis

The IC_50_ values were determined via non-linear regression, using the GraphPad Prism software package version 8 (GraphPad Software, San Diego, CA, USA). Results were expressed as means of IC_50_ values ± SD. The TSI values were calculated using the following formula:

TSI = IC_50_ (treated normal cell line)/IC_50_ (treated tumor cell line)

## 4. Conclusions

A library of fourteen derivatives obtained via the hybridization strategy between DHA **2** and ARTA **3** and monoterpenes **4**–**8** was synthesized. All of these compounds were tested on primary WM215 and metastatic WM266 cell lines using healthy fibroblasts C3PV as reference. Hybrids **9c** and **9f,** deriving from the combination of DHA with citronellol and nerol, and hybrids **10b** and **10f** obtained by linking ARTA to perillyl alcohol and nerol were the best compounds of the series, showing appreciable antimelanoma activity and a moderate cytotoxic effect. These four derivatives were evaluated for their chemical stability in relation to the presence of a removable (ester bond between ARTA and monoterpene) or more stable (ether bond between DHA and monoterpene) linker, obtaining good results from all of them. Studies of coadministration of unfastened sesquiterpene and monoterpene natural products were conducted, unambiguously addressing the importance of the hybridization strategy. Finally, preliminary experiments in the presence of an iron chelator such as DFO showed the dependence of the biological activity of compounds **9c**, **9f**, **10b**, and **10f** on the formation of carbon- and oxygen-centered radical species resulting from the opening of the pharmacophoric sesquiterpene endoperoxide bridge. The hypothesis of a radical-based mode of action is further corroborated by the decrease in the antimelanoma activity obtained with 2-deoxyartemisinin analogues **11c** and **11f** synthesized in this study. Compared to current melanoma chemotherapeutics, the four hybrids described possess IC_50_ values not too far from those of temozolomide, dacarbazine, PTX, and vemurafenib and have certain advantages in terms of cost and synthesis, since they can be obtained in a single step from commercially available products, are safe, are intrinsic to the radical mode of action triggered by iron, and have a low incidence of evoking drug-resistance phenomena.

## Figures and Tables

**Table 1:** Anticancer activity of DHA, ARTA, monoterpenes **4**–**8**, hybrids **9a**–**g** and **10a**–**g**, 2-deoxyartemisin derivatives **11c**,**f,** and Paclitaxel ^a^.

Entry	Compound	S.C. ^b^	IC_50_ (µM ± SD) ^c^			TSI ^d^	
			C3PV	WM115	WM266	WM115	WM266
**1**	DHA **2**	-	0.7 ± 0.19	1.6 ± 0.4	1.6 ± 0.03	0.4	0.4
**2**	ARTA **3**	-	1.7 ± 0.44	1.5 ± 0.01	1.3 ± 0.2	1.1	1.3
**3**	Eugenol **4**	-	1.0 ± 0.1	3.0 ± 0.02	0.9 ± 0.05	0.3	1.1
**4**	Perillyl alcohol **5**	-	52.5 ± 9.5	1.2 ± 0.02	0.6 ± 0.04	43.8	87.5
**5**	(±)-citronellol **(±)-6**	-	3.0 ± 1.2	2.6 ± 0.05	0.3 ± 0.23	1.2	10.0
**6**	(+)-citronellol **(+)-6**	-	1.9 ± 0.8	1.5 ± 0.07	0.6 ± 0.02	1.3	3.2
**7**	(-)-citronellol **(-)-6**	-	1.0 ± 0.9	0.9 ± 0.03	0.5 ± 0.3	1.1	2.0
**8**	Nerol **7**	-	3.8 ± 1.5	1.4 ± 0.03	0.5 ± 0.01	2.7	7.6
**9**	Geraniol **8**	-	0.5 ± 0.02	0.5 ± 0.01	0.5 ± 0.09	1.0	1.0
**10**	**9a**	DHA	0.7 ± 0.04	0.1 ± 0.01	1.0 ± 0.09	7.0	0.7
**11**	**9b**	DHA	0.3 ± 0.1	0.2 ± 0.01	0.2 ± 0.01	1.5	1.5
**12**	**9c**	DHA	364.2 ± 7.9	2.1 ± 0.3	1.4 ± 0.56	173.4	260.1
**13**	**9d**	DHA	51.0 ± 0.3	2.9 ± 0.6	2.7 ± 0.2	17.6	18.9
**14**	**9e**	DHA	50.0 ± 0.03	2.4 ± 0.1	2.2 ± 0.6	20.8	22.7
**15**	**9f**	DHA	87.3 ± 2.5	3.0 ± 0.4	1.9 ± 0.5	29.1	45.9
**16**	**9g**	DHA	6.2 ± 0.7	14.5 ± 1.1	13.4 ± 1.5	0.4	0.5
**17**	**10a**	ARTA	1.6 ± 0.3	1.5 ± 0.8	0.6 ± 0.1	1.1	2.7
**18**	**10b**	ARTA	20.3 ± 5.5	0.03 ± 0.01	0.02 ± 0.01	676.7	1015.0
**19**	**10c**	ARTA	4.4 ± 2.9	1.3 ± 0.9	0.6 ± 0.02	3.4	7.3
**20**	**10d**	ARTA	4.8 ± 0.9	1.7 ± 0.5	1.3 ± 0.8	2.8	3.7
**21**	**10e**	ARTA	5.1 ± 1.7	1.9 ± 0.6	1.6 ± 0.9	2.7	3.2
**22**	**10f**	ARTA	7.9 ± 4.5	0.4 ± 0.04	0.09 ± 0.03	19.8	87.8
**23**	**10g**	ARTA	2.4 ± 0.9	1.5 ± 0.7	1.0 ± 0.1	1.6	2.4
**24**	**PTX**	-	78.9 ± 0.8	2.3 ± 0.7	0.9 ± 0.04	34.3	87.7
**25**	**11c**	2-dDHA ^e^	-	-	35.1 ± 2.5	-	-
**26**	**11f**	2-dDHA ^e^	-	-	76.3 ± 9.5	-	-

^a^ All experiments were performed in triplicates. ^b^ S.C. = sesquiterpene counterpart. ^c^ IC_50_ ± SD (half-maximal inhibitory concentration ± standard deviation) values for all compounds are expressed in μM units. ^d^ TSI (tumor selectivity index) obtained as the ratio between the IC_50_ value on C3PV and the IC_50_ value on WM115 and WM266 cell lines, using the following formula: IC_50_ (treated wt cell line)/IC_50_ (treated tumor cell line). ^e^ 2-dDHA = 2-deoxydihydroartemisinin.

**Table 2:** Antimelanoma effect evaluated after co-administration of unfastened parent compounds of hybrids **9c**, **9f**, **10b**, and **10f** ^a^.

Entry	Combination	R.H. ^b^	IC_50_ ± SD WM266 ^c^	IC_50_ ± SD R.H. ^d^
**1**	DHA+citronellol **(±)-6**	**9c**	4.56 ± 07	1.4 ± 0.56
**2**	DHA+nerol **7**	**9f**	5.49 ± 0.6	1.9 ± 0.5
**3**	ARTA+perillyl alcohol **5**	**10b**	1.8 ± 0.3	0.02 ± 0.01
**4**	ARTA+nerol **7**	**10f**	2.9 ± 0.9	0.09 ± 0.03

^a^ All experiments were performed in triplicates. ^b^ R.H. = reference hybrid. ^c^ IC_50_ ± SD (half-maximal inhibitory concentration ± standard deviation) values on WM266 cell line expressed in μM units. ^d^ IC_50_ ± SD values on WM266 cell line expressed in μM units of the reference hybrid.

**Table 3:** Cell viability assay in the presence or absence of DFO ^a^.

Entry	Compound ^b^	IC_50_ ± SD WM266 ^b,c^	
		without DFO	with DFO
**1**	**9c**	1.4 ± 0.56	12.7 ± 0.3
**2**	**9f**	1.9 ± 0.5	132.7 ± 0.4
**3**	**10b**	0.02 ± 0.01	0.31 ± 0.1
**4**	**10f**	0.09 ± 0.03	0.5 ± 0.1

^a^ All experiments were performed in triplicates. ^b^ The treatment time was 48 h for all experiments. ^c^ IC_50_ ± SD (half-maximal inhibitory concentration ± standard deviation) values for all compounds are expressed in μM units.

## Data Availability

Data are contained within the article and Supplementary Materials.

## Supplementary Materials

The following supporting information can be downloaded at https://www.mdpi.com/article/10.3390/molecules29143421/s1, S1: NMR stability experiments title; S2: ^1^H, ^13^C NMR, and IR spectra.
