# Supplementary material for: Modulation of the Antimelanoma Activity Imparted to Artemisinin Hybrids by the Monoterpene Counterpart
Source: Molecules. 2024 Jul 21;29(14):3421. doi: 10.3390/molecules29143421 (PMC11279807; doi:10.3390/molecules29143421)

**Supplementary materials for**

# Modulation of the Antimelanoma Activity Imparted to Artemisinin Hybrids by the Monoterpene Counterpart

**Elisa De Marchi [1], Silvia Filippi [1], Silvia Cesarini [1], Beatrice Di Maio [1], Bruno Mattia Bizzarri [1], Raffaele Saladino [1] and Lorenzo Botta [1,*]**

[1] Department of Biological and Ecological Sciences, University of Viterbo, Via S.C. De Lellis s.n.c., 01100 Viterbo, Italy; elisa.demarchi@unitus.it; silvia.filippi@unitus.it; c.cesarinisilvia@gmail.com; beatrice.dimaio@studenti.unitus.it; bm.bizzarri@unitus.it; saladino@unitus.it

* Correspondence: lorenzo.botta@unitus.it; Tel.: +39 0761-357206

**Table of contents**

**S1. Stability Experiments**

**S2. $^{1}H$, $^{13}C$ NMR and IR spectra**

## S1. Stability Experiments

Stability experiments of hybrid compounds **9c**, **9f**, **10b** and **10f** were performed by $^{1}$H-NMR analysis on a Bruker Avance DRX400 (400 MHz). The selected hybrids were dissolved (8-9 mg) in $CDCl_3$ and the NMR tubes heated for at least 8 h at 45 °C in an oil bath. The same NMR tubes were then heated for at least 2 h at 100 °C.

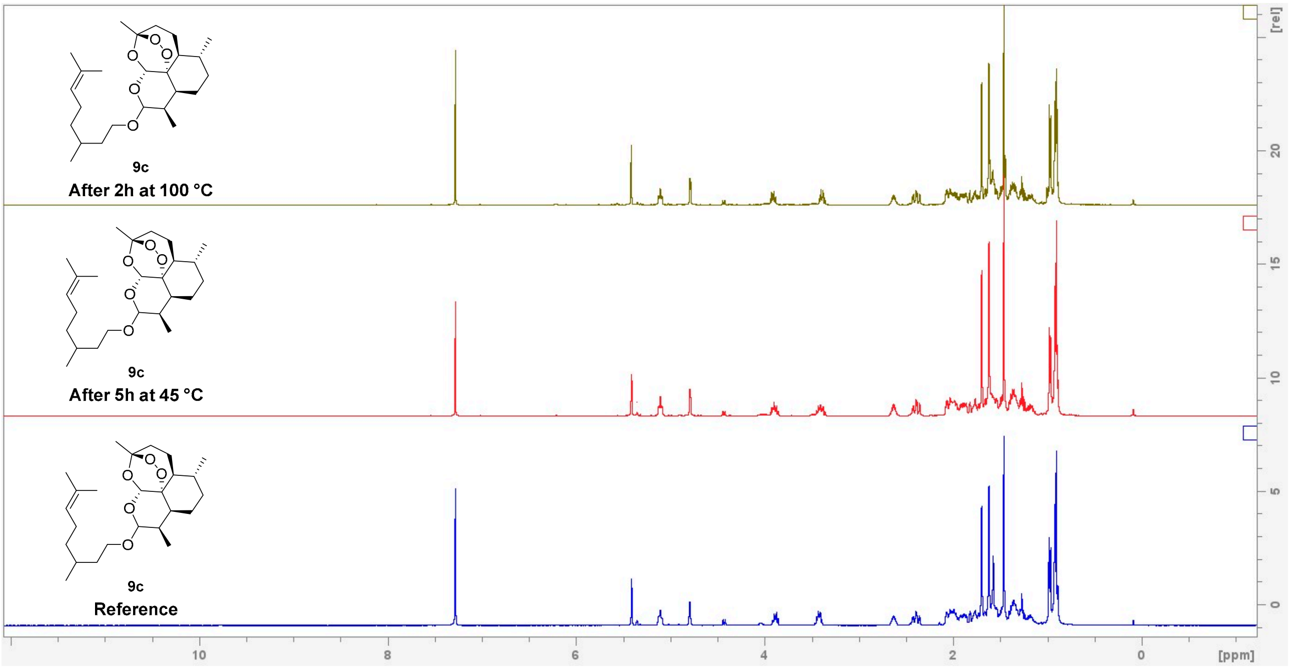


**Figure S1:** $^{1}$H NMR spectra of hybrid **9c** reference (bottom, coloured blue), after 5h at 45 °C (middle, coloured red) and after 2h at 100 °C (top, coloured gold).

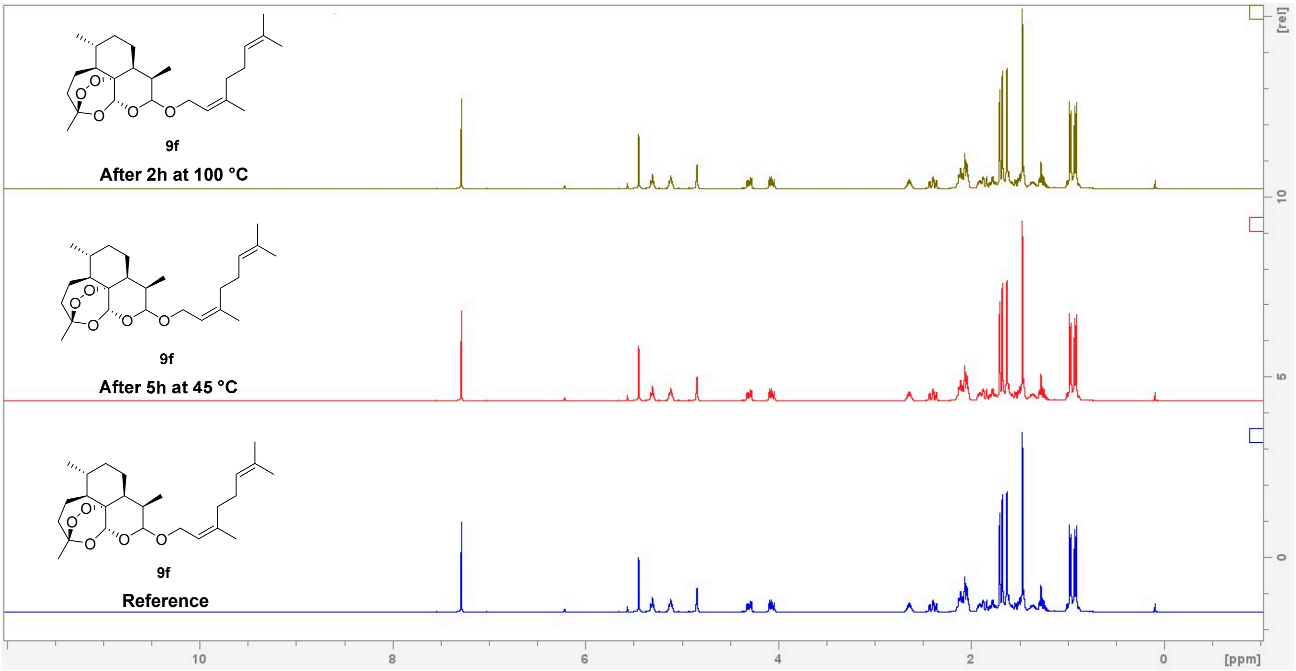


**Figure S2:** $^{1}$H NMR spectra of hybrid **9f** reference (bottom, coloured blue), after 5h at 45 °C (middle, coloured red) and after 2h at 100 °C (top, coloured gold).

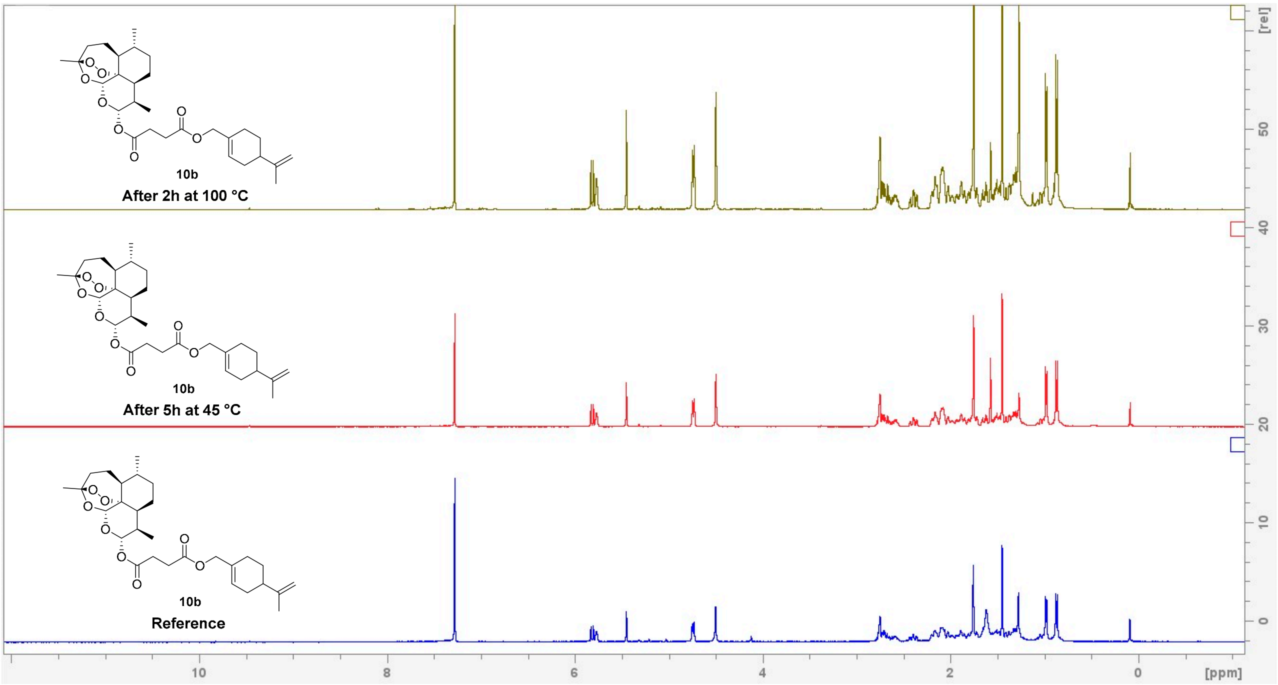


**Figure S3:** $^{1}$H NMR spectra of hybrid **10b** reference (bottom, coloured blue), after 5h at 45 °C (middle, coloured red) and after 2h at 100 °C (top, coloured gold).

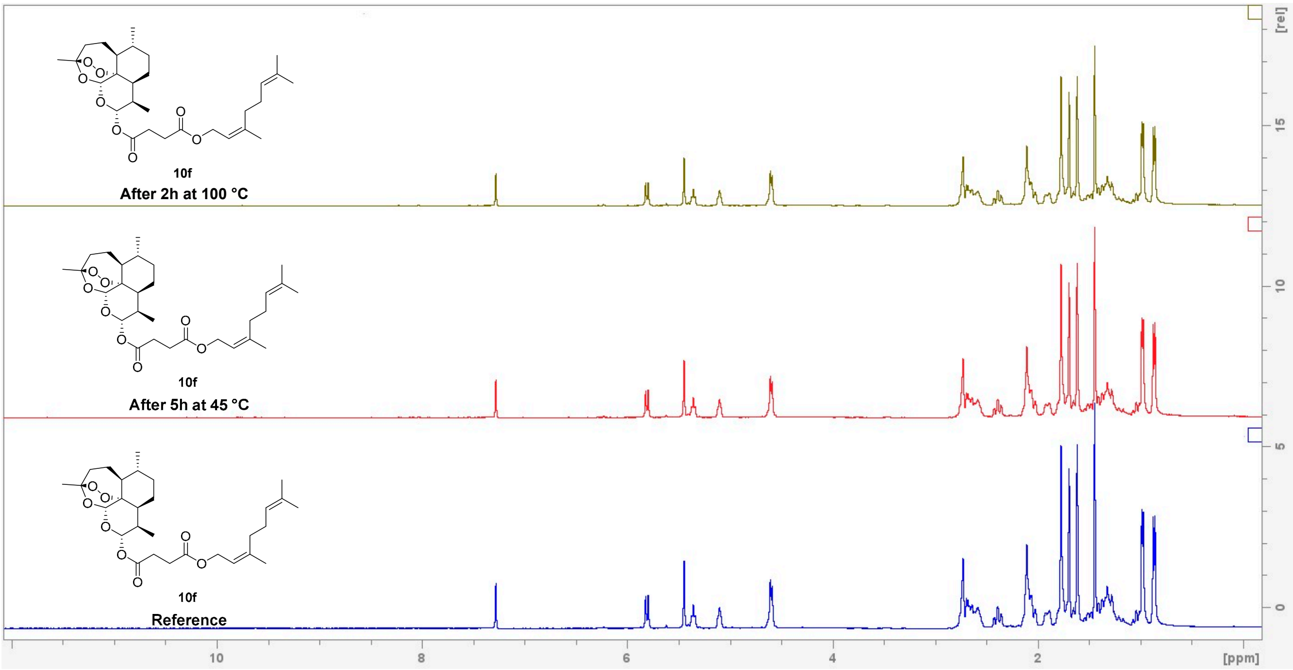


**Figure S4:** $^{1}$H NMR spectra of hybrid **10f** reference (bottom, coloured blue), after 5h at 45 °C (middle, coloured red) and after 2h at 100 °C (top, coloured gold).

## S2. $^{1}H$, $^{13}C$ NMR and IR spectra

### *Hybrid 9a*

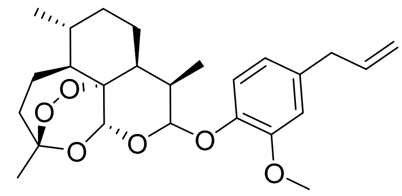

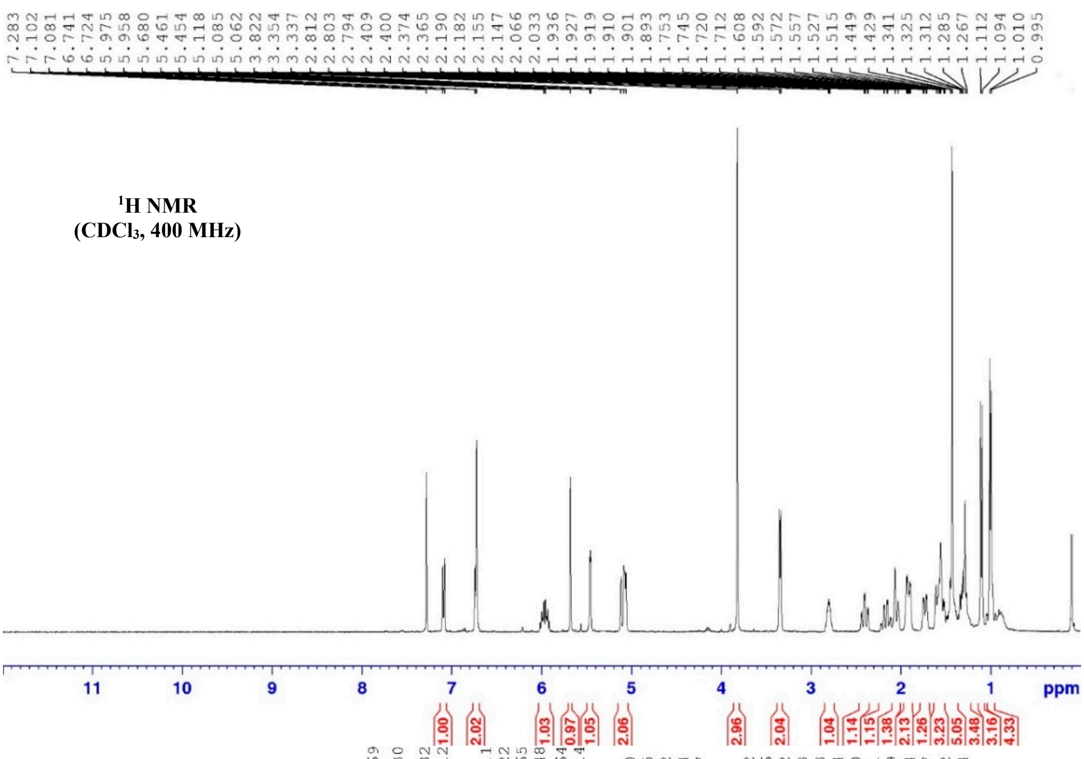


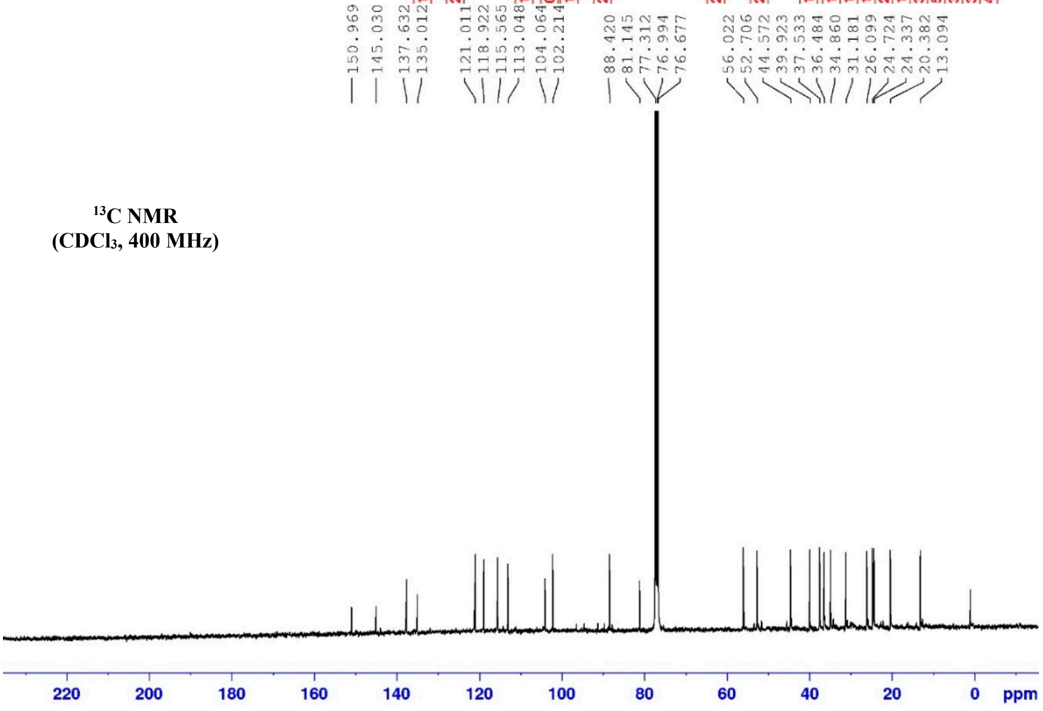

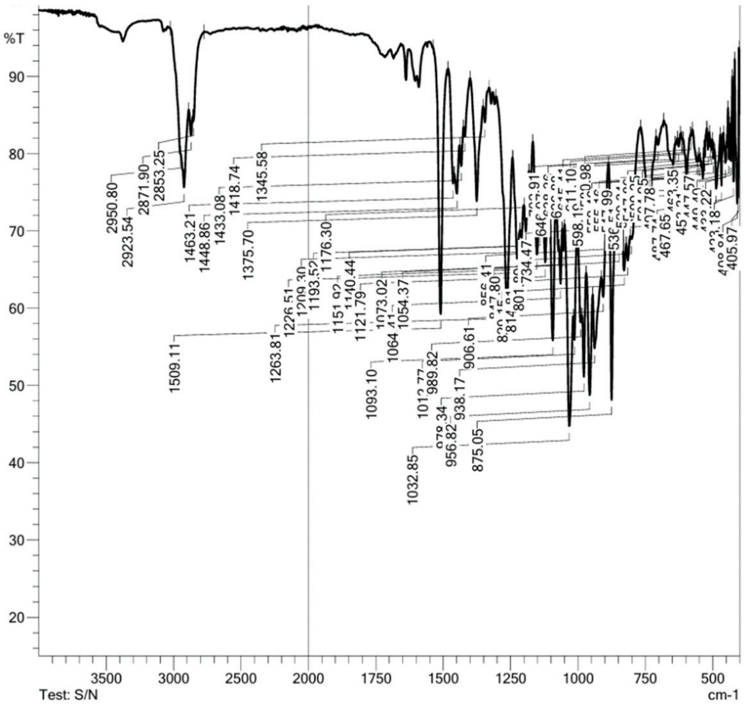
%T
Test: S/N
cm-1

***Hybrid 9b***

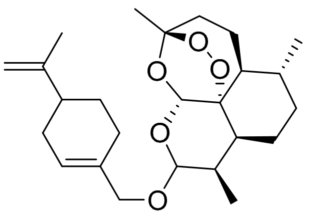

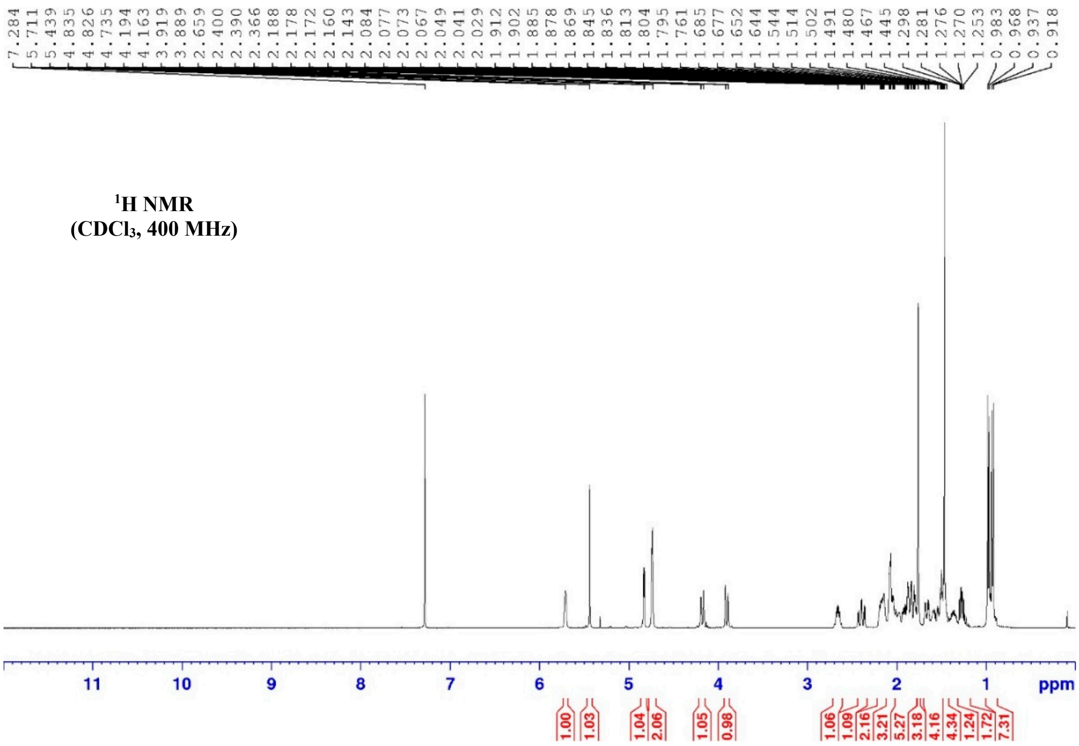
7.284
5.711
5.439
4.835
4.826
4.735
4.194
4.163
3.919
3.889
2.659
2.400
2.390
2.366
2.188
2.178
2.172
2.160
2.143
2.084
2.077
2.073
2.067
2.049
2.041
2.029
1.912
1.902
1.885
1.878
1.869
1.845
1.836
1.813
1.804
1.795
1.761
1.685
1.677
1.652
1.644
1.544
1.514
1.502
1.491
1.480
1.467
1.445
1.298
1.281
1.276
1.270
1.253
0.983
0.968
0.937
0.918
$^1$H NMR
(CDCl$_3$, 400 MHz)
11
10
9
8
7
6
5
4
3
2
1
ppm
1.00
1.03
1.04
2.06
1.05
0.98
1.06
1.09
2.16
3.21
5.27
3.18
4.16
4.34
1.24
1.72
7.31

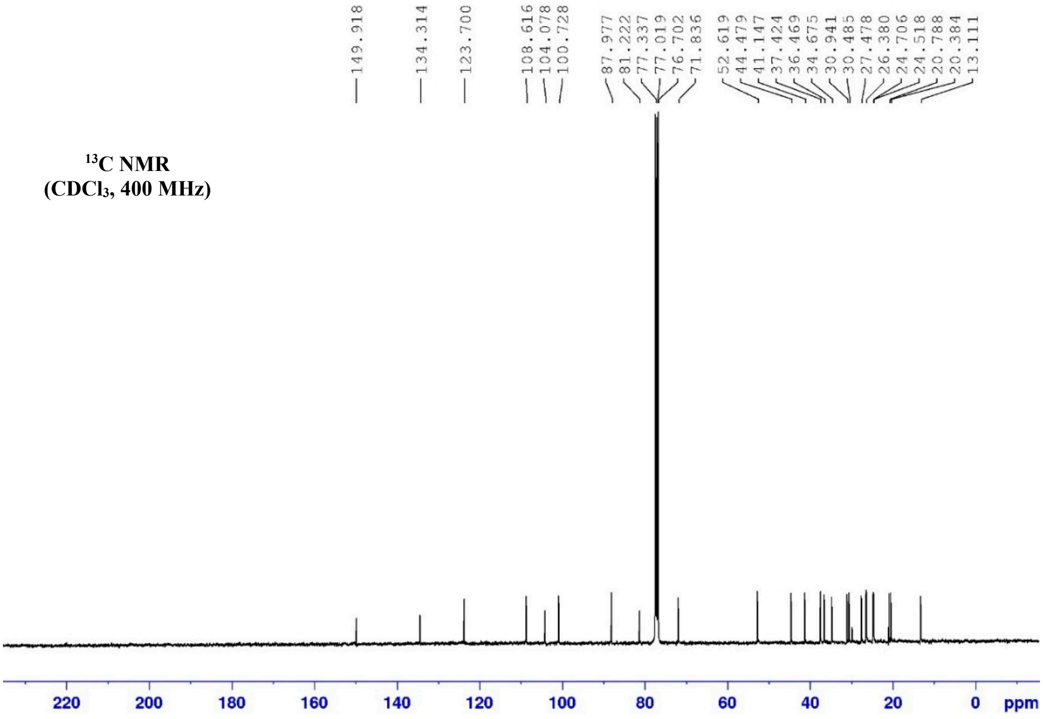
149.918
134.314
123.700
108.616
104.078
100.728
87.977
81.222
77.337
77.019
76.702
71.836
52.619
44.479
41.147
37.424
36.469
34.675
30.941
30.485
27.478
26.380
24.706
24.518
20.788
20.384
13.111
$^{13}$C NMR
(CDCl$_3$, 400 MHz)
220
200
180
160
140
120
100
80
60
40
20
0
ppm

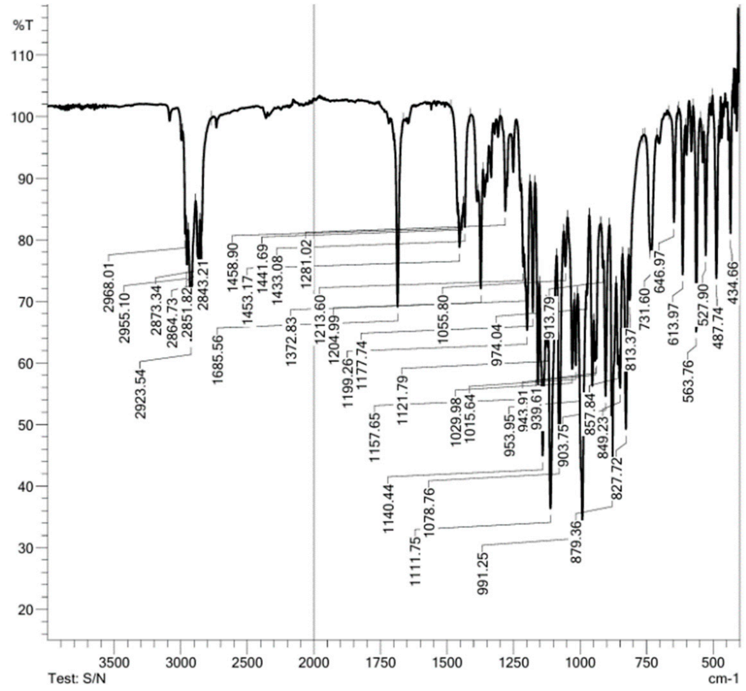

%T
110
100
90
80
70
60
50
40
30
20
2968.01
2955.10
2873.34
2864.73
2851.82
2843.21
2923.54
1685.56
1458.90
1453.17
1441.69
1433.08
1372.83
1281.02
1213.60
1204.99
1199.26
1177.74
1157.65
1140.44
1121.79
1111.75
1078.76
1055.80
1029.98
1015.64
991.25
974.04
953.95
943.91
939.61
913.79
903.75
879.36
857.84
849.23
827.72
813.37
731.60
646.97
613.97
563.76
527.90
487.74
434.66
3500
3000
2500
2000
1750
1500
1250
1000
750
500
Test: S/N
cm-1

***Hybrid 9c***

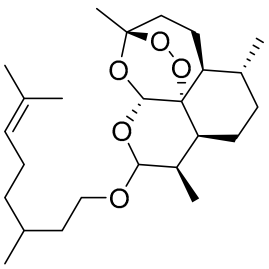
O
O
O
O
O

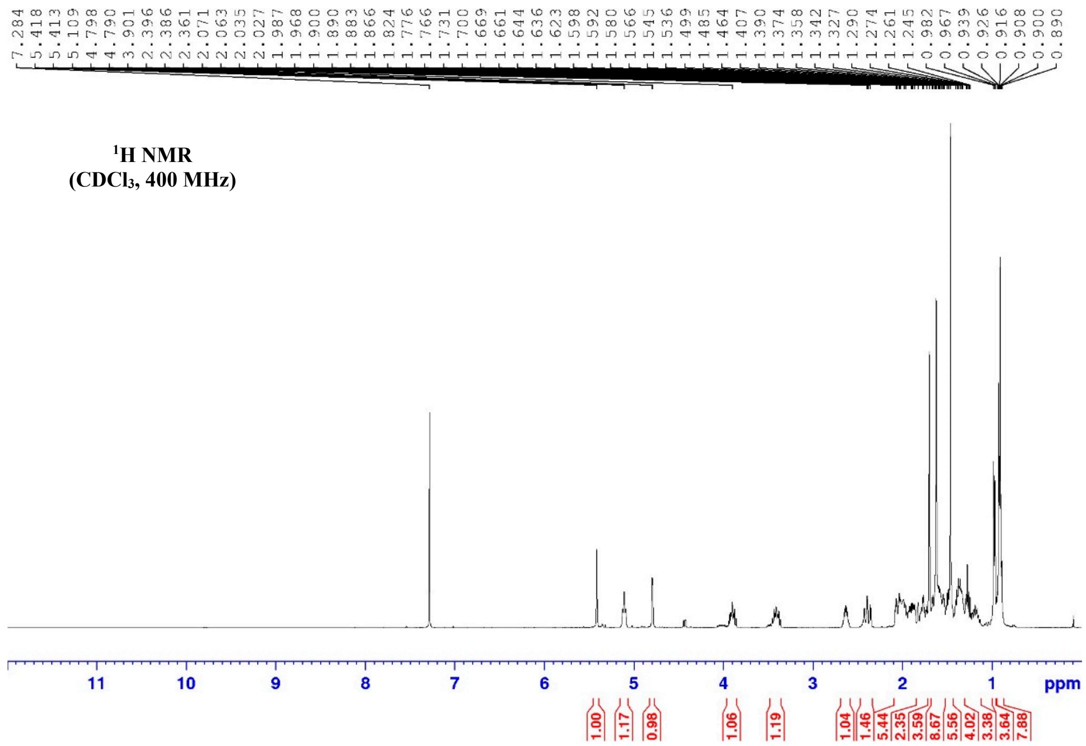
7.284
5.418
5.413
5.109
4.798
4.790
3.901
2.396
2.386
2.361
2.071
2.063
2.035
2.027
1.987
1.968
1.900
1.890
1.883
1.866
1.824
1.776
1.766
1.731
1.700
1.669
1.661
1.644
1.636
1.623
1.598
1.592
1.580
1.566
1.545
1.536
1.499
1.485
1.464
1.407
1.390
1.374
1.358
1.342
1.327
1.290
1.274
1.261
1.245
0.982
0.967
0.939
0.926
0.916
0.908
0.900
0.890
¹H NMR
(CDCl₃, 400 MHz)
11
10
9
8
7
6
5
4
3
2
1
ppm
1.00
1.17
0.98
1.06
1.19
1.04
1.46
5.44
2.35
3.59
8.67
5.56
4.02
3.38
3.64
7.88

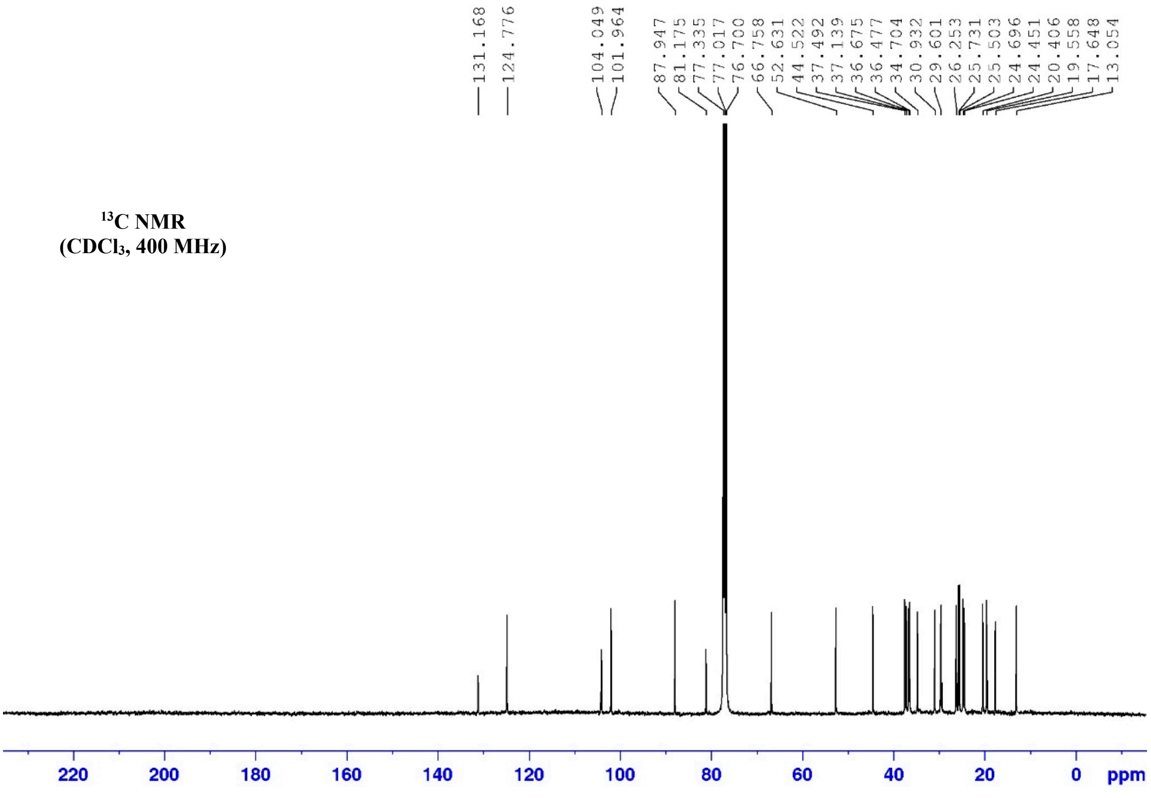
131.168
124.776
104.049
101.964
87.947
81.175
77.335
77.017
76.700
66.758
52.631
44.522
37.492
37.139
36.675
36.477
34.704
30.932
29.601
26.253
25.731
25.503
24.696
24.451
20.406
19.558
17.648
13.054
¹³C NMR
(CDCl₃, 400 MHz)
220
200
180
160
140
120
100
80
60
40
20
0
ppm

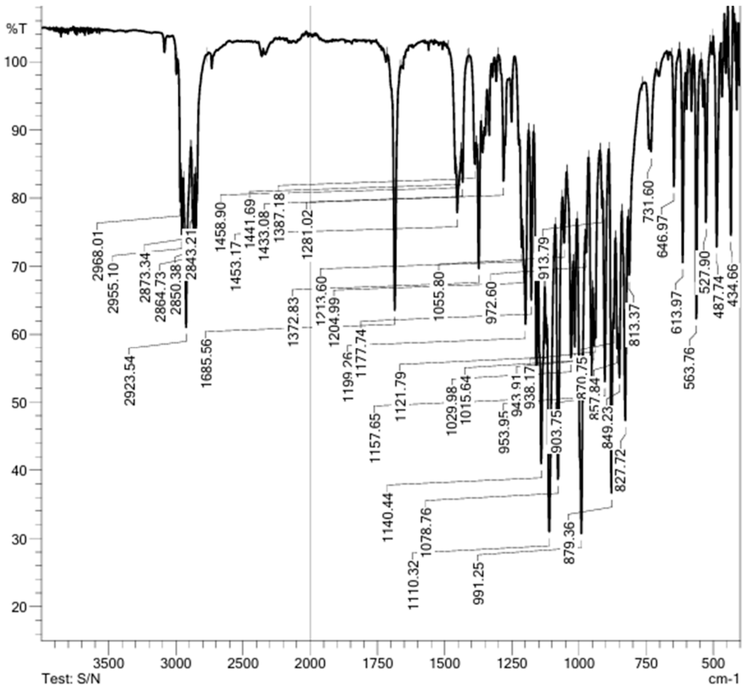
%T
100
90
80
70
60
50
40
30
20
3500
3000
2500
2000
1750
1500
1250
1000
750
500
Test: S/N
cm-1
2968.01
2955.10
2873.34
2864.73
2850.38
2843.21
2923.54
1685.56
1458.90
1453.17
1441.69
1433.08
1387.18
1372.83
1281.02
1213.60
1204.99
1199.26
1177.74
1157.65
1140.44
1121.79
1110.32
1078.76
1055.80
1029.98
1015.64
991.25
972.60
953.95
943.91
938.17
913.79
903.75
879.36
870.75
857.84
849.23
827.72
813.37
731.60
646.97
613.97
563.76
527.90
487.74
434.66

*Hybrid 9d*

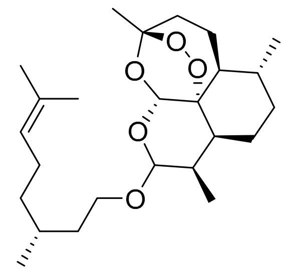

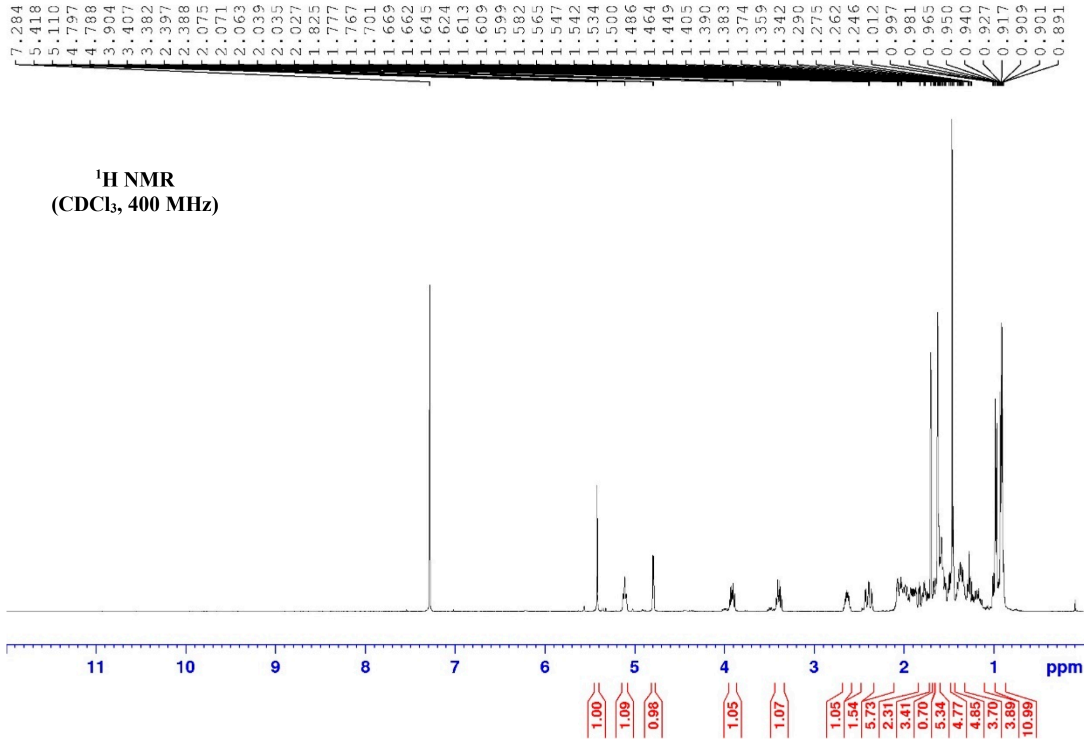

7.284
5.418
5.110
4.797
4.788
3.904
3.407
3.382
2.397
2.388
2.075
2.071
2.063
2.039
2.035
2.027
1.825
1.777
1.767
1.701
1.669
1.662
1.645
1.624
1.613
1.609
1.599
1.582
1.565
1.547
1.542
1.534
1.500
1.486
1.464
1.449
1.405
1.390
1.383
1.374
1.359
1.342
1.290
1.275
1.262
1.246
1.012
0.997
0.981
0.965
0.950
0.940
0.927
0.917
0.909
0.901
0.891
1H NMR
(CDCl3, 400 MHz)
11
10
9
8
7
6
5
4
3
2
1
ppm
1.00
1.09
0.98
1.05
1.07
1.05
1.54
5.73
2.31
3.41
0.70
5.34
4.77
4.85
3.70
3.89
10.99


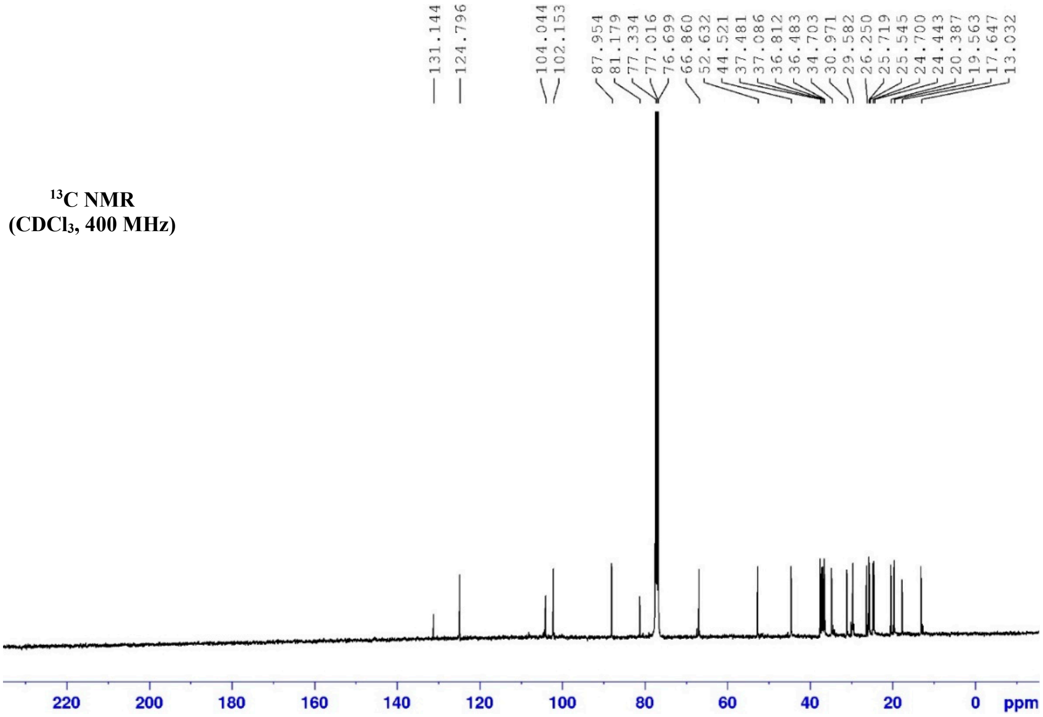

131.144
124.796
104.044
102.153
87.954
81.179
77.334
77.016
76.699
66.860
52.632
44.521
37.481
37.086
36.812
36.483
34.703
30.971
29.582
26.250
25.719
25.545
24.700
24.443
20.387
19.563
17.647
13.032
13C NMR
(CDCl3, 400 MHz)
220
200
180
160
140
120
100
80
60
40
20
0
ppm

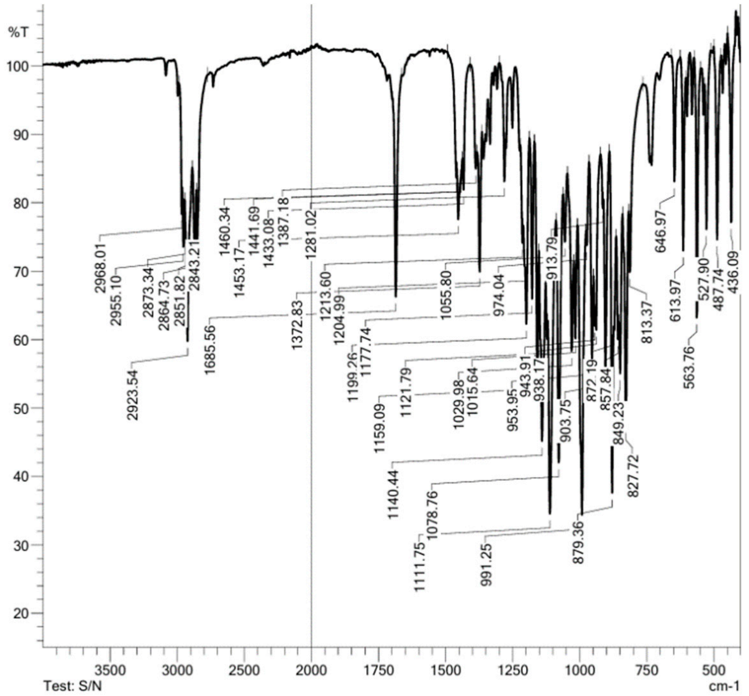
%T
Test: S/N
cm-1
2968.01
2955.10
2873.34
2864.73
2851.82
2843.21
2923.54
1685.56
1460.34
1453.17
1441.69
1433.08
1387.18
1372.83
1281.02
1213.60
1204.99
1199.26
1177.74
1159.09
1140.44
1121.79
1111.75
1078.76
1055.80
1029.98
1015.64
991.25
974.04
953.95
943.91
938.17
913.79
903.75
879.36
872.19
857.84
849.23
827.72
813.37
646.97
613.97
563.76
527.90
487.74
436.09

***Hybrid 9e***

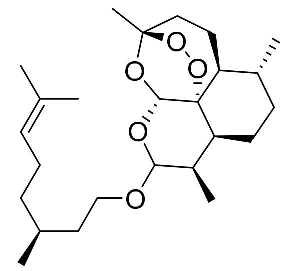

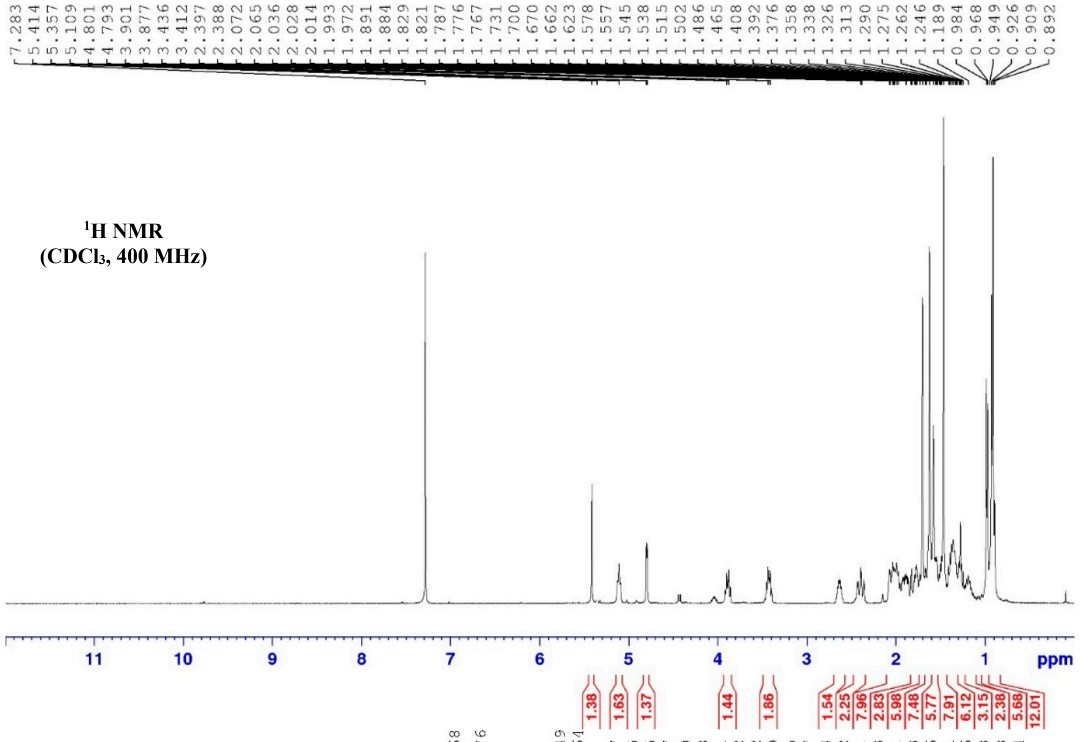

7.283
5.414
5.357
5.109
4.801
4.793
3.901
3.877
3.436
3.412
2.397
2.388
2.072
2.065
2.036
2.028
2.014
1.993
1.972
1.891
1.884
1.829
1.821
1.787
1.776
1.767
1.731
1.700
1.670
1.662
1.623
1.578
1.557
1.545
1.538
1.515
1.502
1.486
1.465
1.408
1.392
1.376
1.358
1.338
1.326
1.313
1.290
1.275
1.262
1.246
1.189
0.984
0.968
0.949
0.926
0.909
0.892
$^{1}$H NMR
($CDCl_3$, 400 MHz)
11
10
9
8
7
6
5
4
3
2
1
ppm
1.38
1.63
1.37
1.44
1.86
1.54
2.25
7.96
2.83
5.98
7.48
5.77
7.91
6.12
3.15
2.38
5.68
12.01


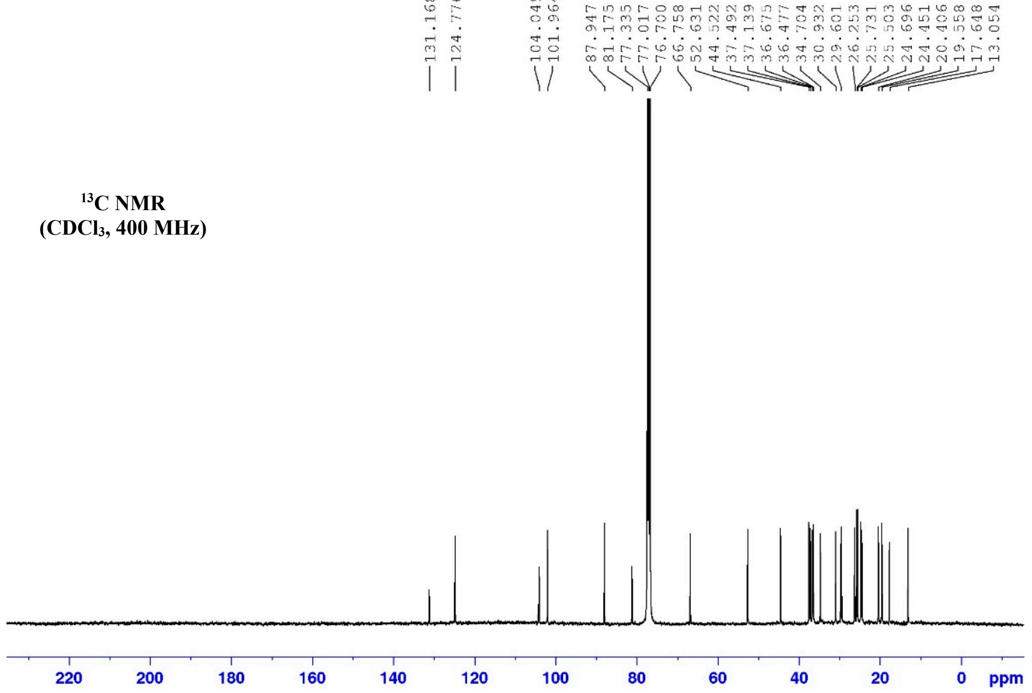

131.168
124.776
104.049
101.964
87.947
81.175
77.335
77.017
76.700
66.758
52.631
44.522
37.492
37.139
36.675
36.477
34.704
30.932
29.601
26.253
25.731
25.503
24.696
24.451
20.406
19.558
17.648
13.054
$^{13}$C NMR
($CDCl_3$, 400 MHz)
220
200
180
160
140
120
100
80
60
40
20
0
ppm

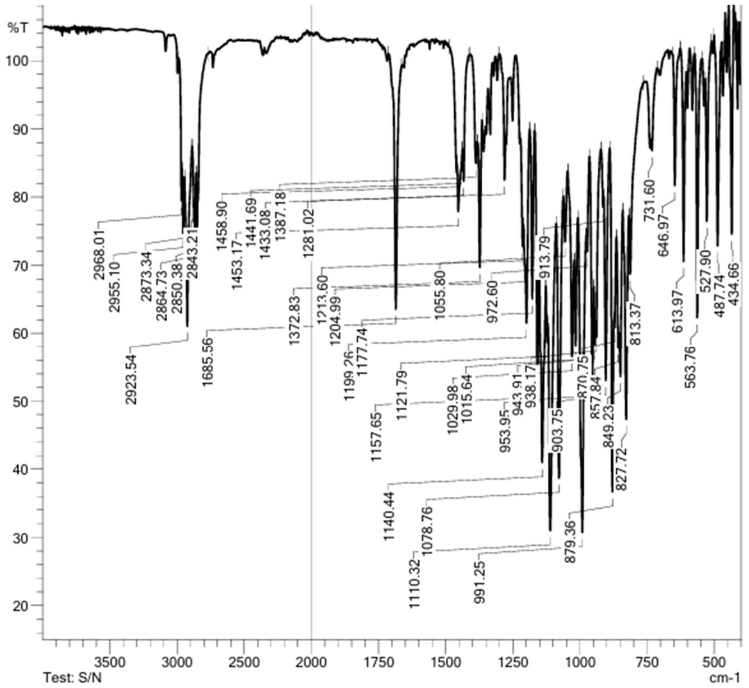
%T
Test: S/N
cm-1
2968.01
2955.10
2873.34
2864.73
2850.38
2843.21
2923.54
1458.90
1453.17
1441.69
1433.08
1387.18
1281.02
1685.56
1372.83
1213.60
1204.99
1199.26
1177.74
1157.65
1121.79
1140.44
1078.76
1110.32
1055.80
1029.98
1015.64
972.60
953.95
943.91
938.17
913.79
903.75
991.25
879.36
870.75
857.84
849.23
827.72
813.37
731.60
646.97
613.97
563.76
527.90
487.74
434.66

*Hybrid 9f*

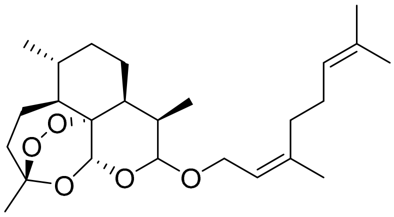

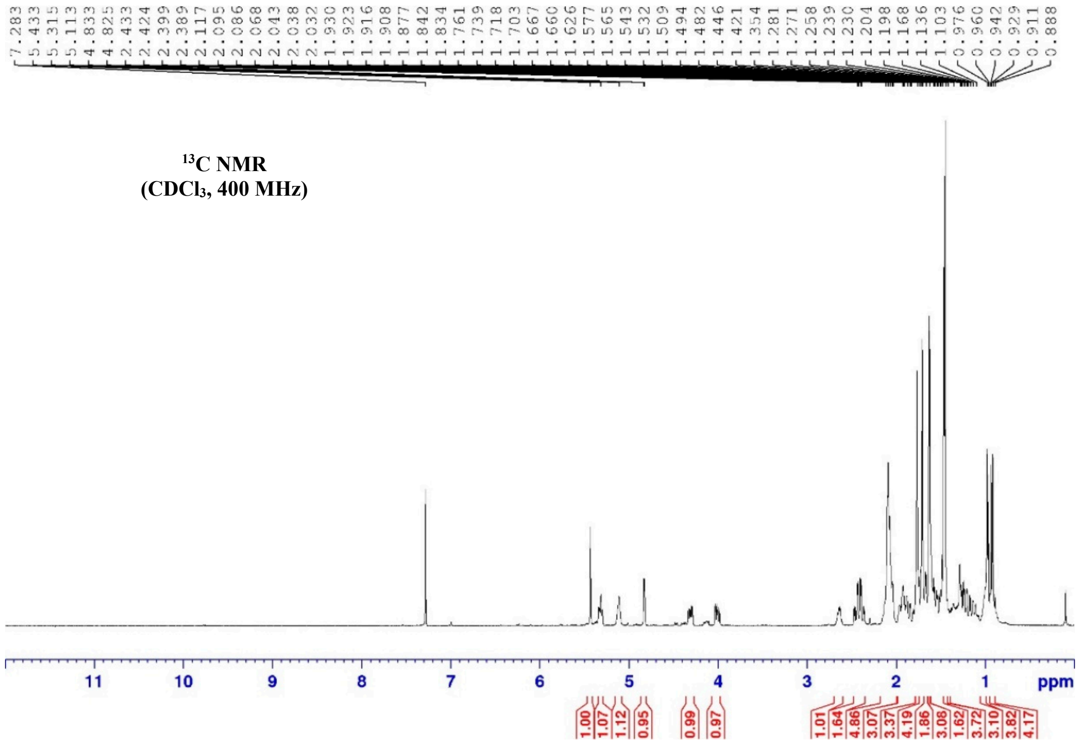


**$^{13}$C NMR**
**($CDCl_3$, 400 MHz)**

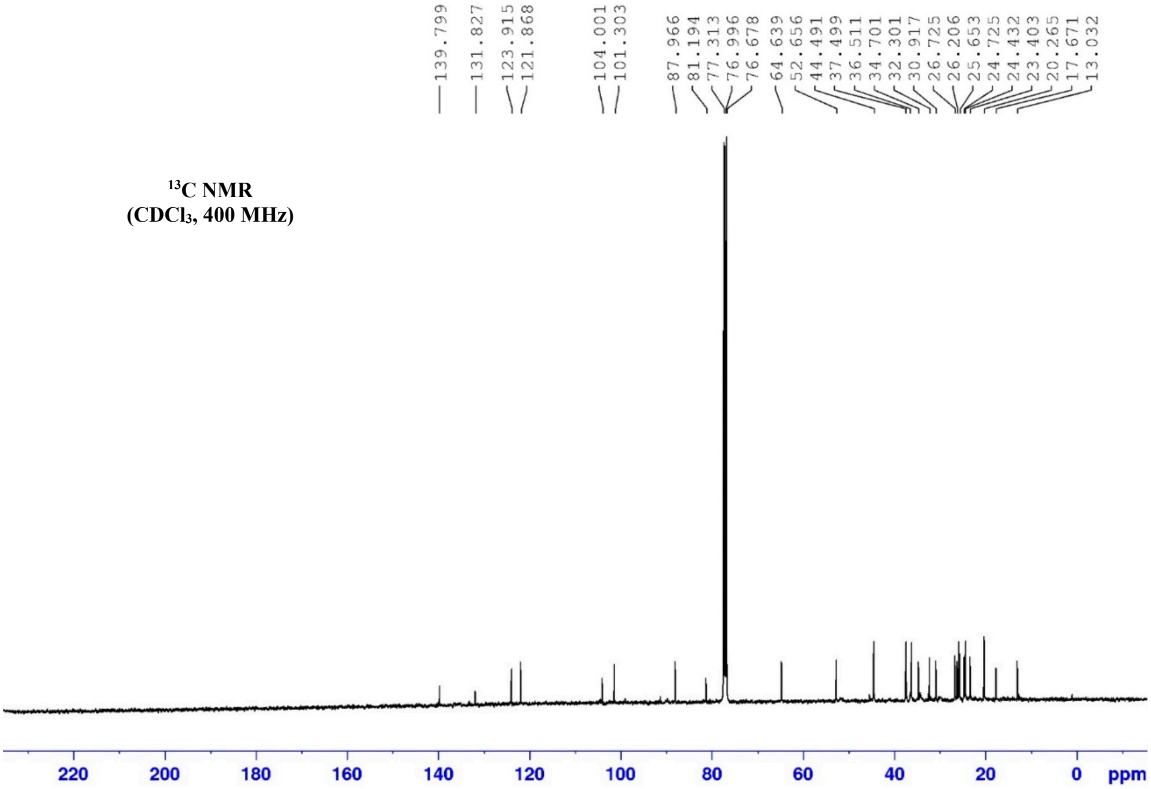


**$^{13}$C NMR**
**($CDCl_3$, 400 MHz)**

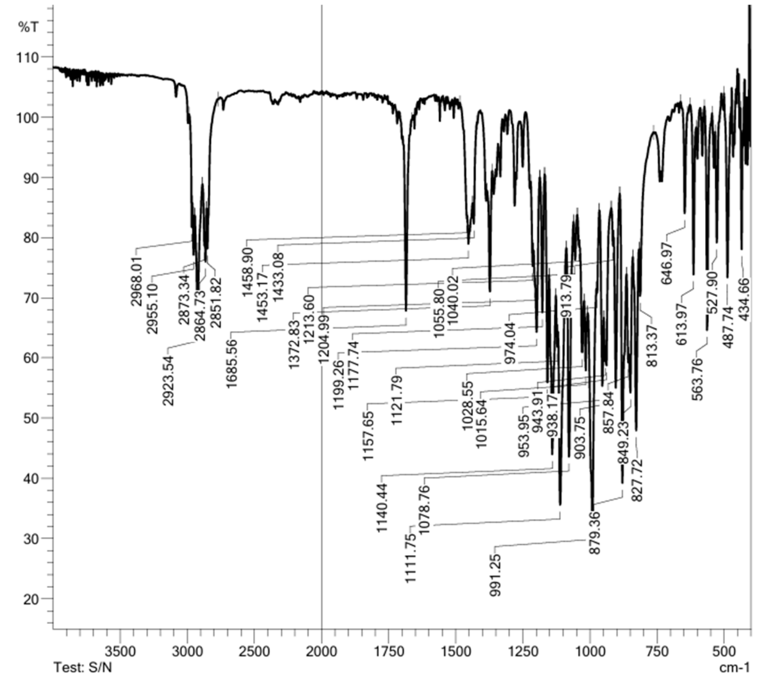
%T
110
100
90
80
70
60
50
40
30
20
3500
3000
2500
2000
1750
1500
1250
1000
750
500
Test: S/N
cm-1
2968.01
2955.10
2923.54
2873.34
2864.73
2851.82
1685.56
1458.90
1453.17
1433.08
1372.83
1213.60
1204.99
1199.26
1177.74
1157.65
1121.79
1140.44
1111.75
1078.76
1055.80
1040.02
1028.55
1015.64
991.25
974.04
953.95
943.91
938.17
913.79
903.75
879.36
857.84
849.23
827.72
813.37
646.97
613.97
563.76
527.90
487.74
434.66

***Hybrid 9g***

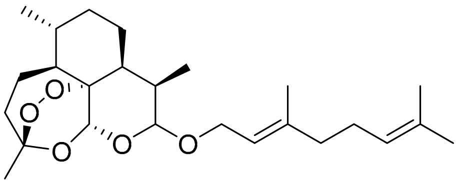


$^1$H NMR
(CDCl$_3$, 400 MHz)

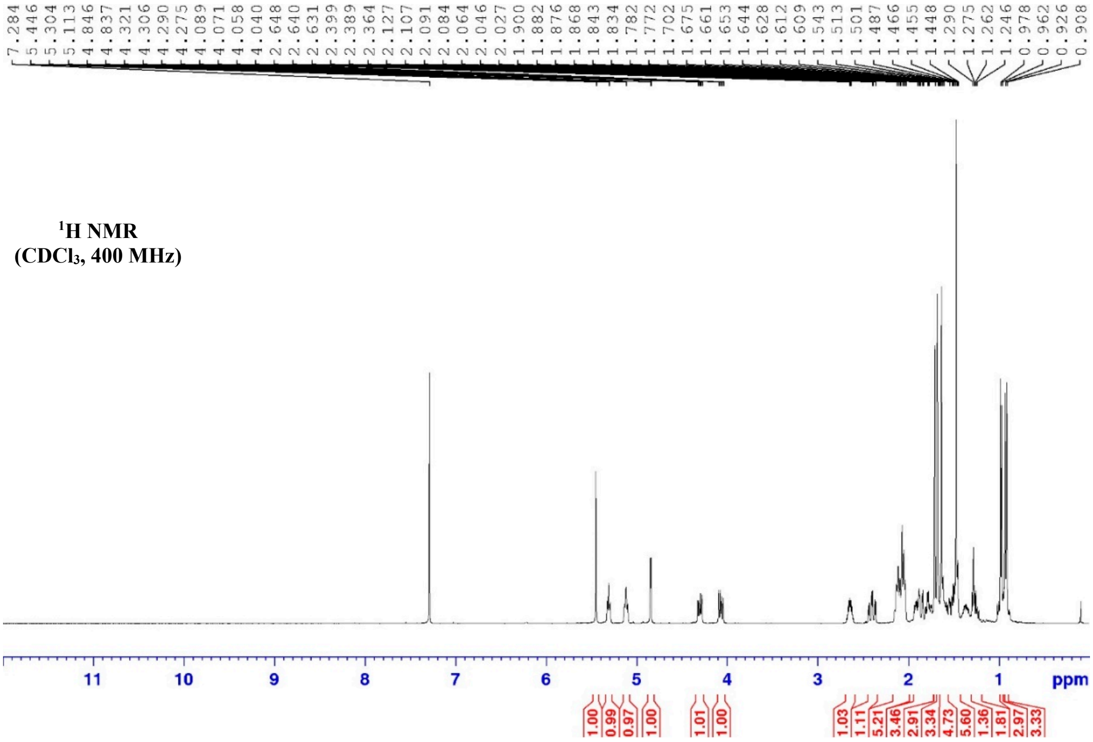


$^{13}$C NMR
(CDCl$_3$, 400 MHz)

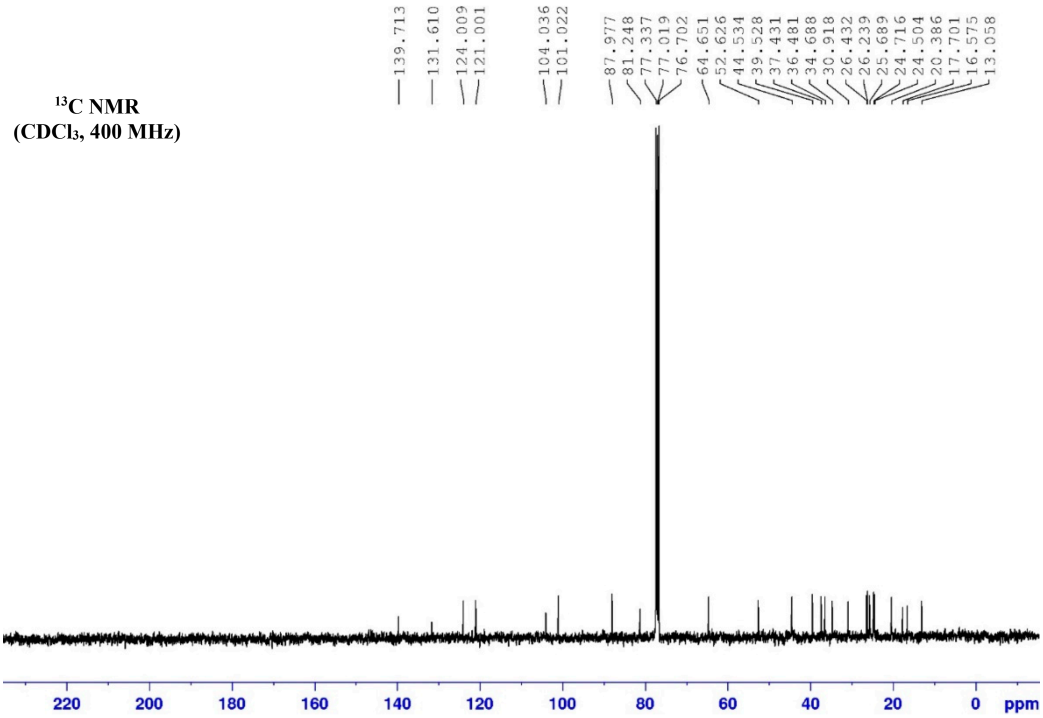

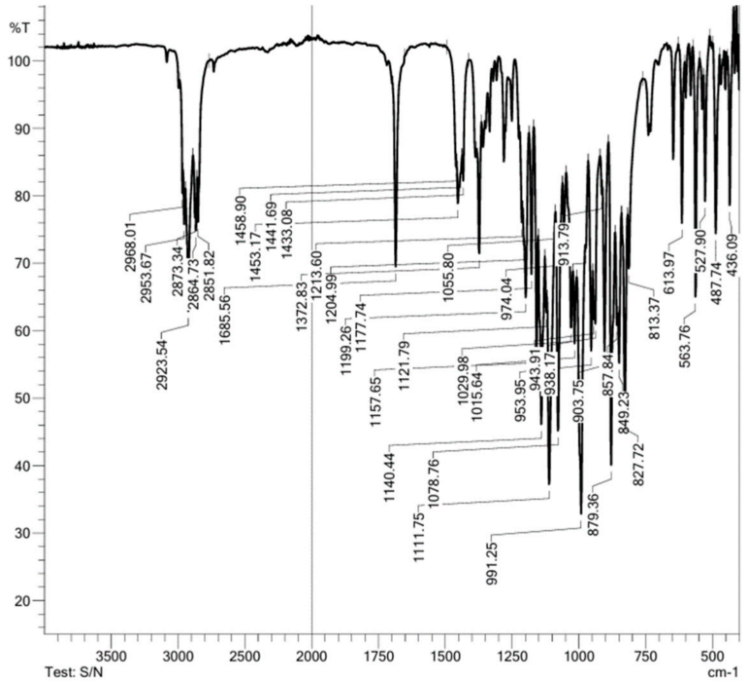

%T
100
90
80
70
60
50
40
30
20
3500
3000
2500
2000
1750
1500
1250
1000
750
500
Test: S/N
cm-1
2968.01
2953.67
2873.34
2864.73
2851.82
2923.54
1458.90
1453.17
1441.69
1433.08
1685.56
1372.83
1213.60
1204.99
1199.26
1177.74
1157.65
1121.79
1055.80
1029.98
1015.64
974.04
953.95
943.91
938.17
913.79
903.75
857.84
849.23
827.72
813.37
1140.44
1078.76
1111.75
991.25
879.36
613.97
563.76
527.90
487.74
436.09

***Hybrid 10a***

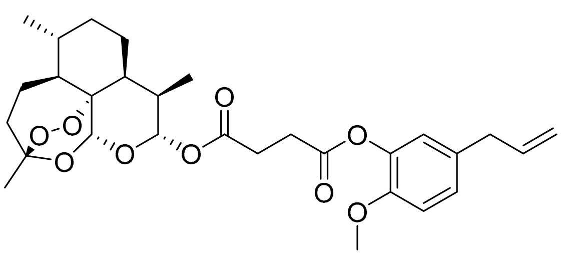

$^{1}$H NMR
(CDCl$_3$, 400 MHz)

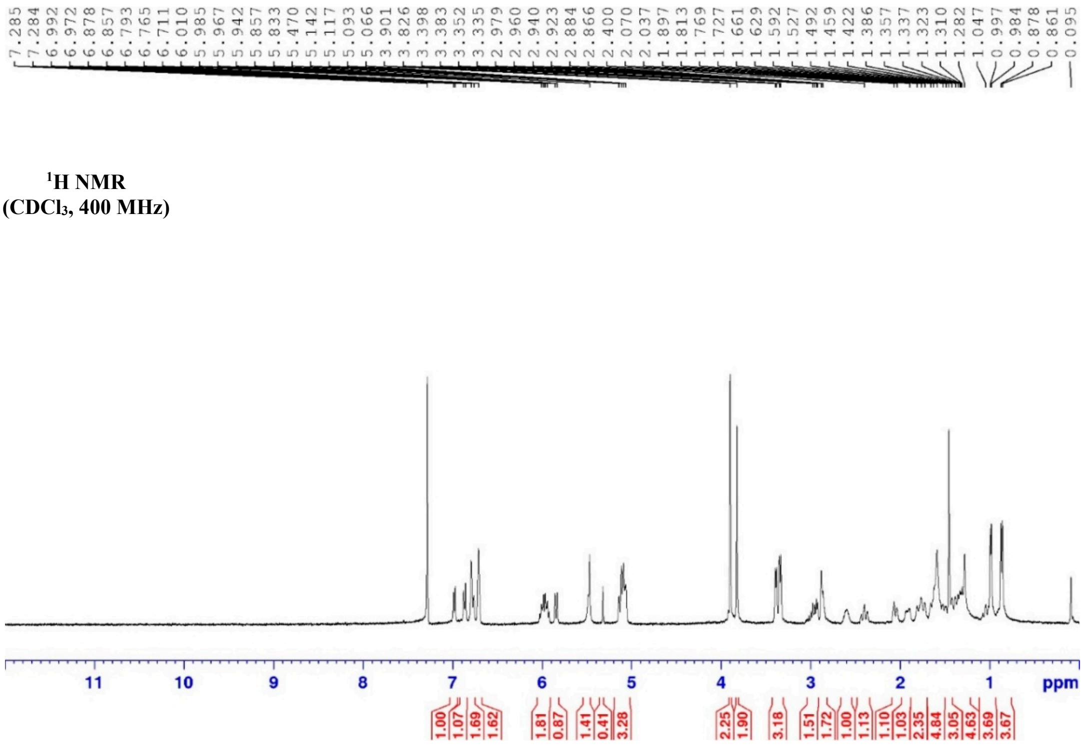


$^{13}$C NMR
(CDCl$_3$, 400 MHz)

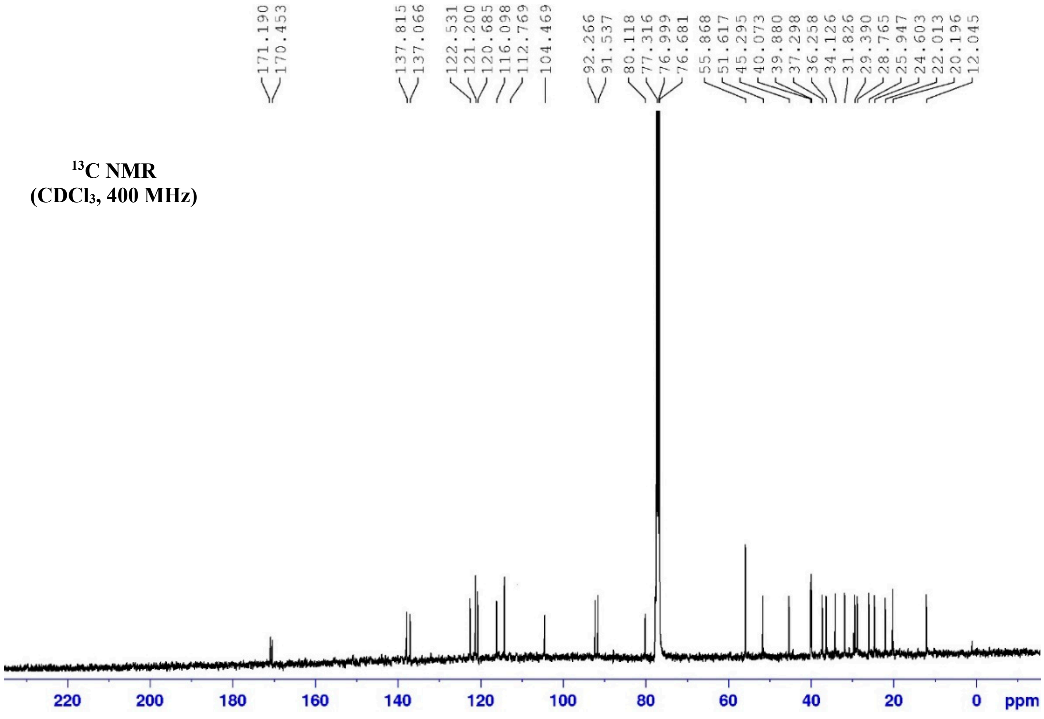

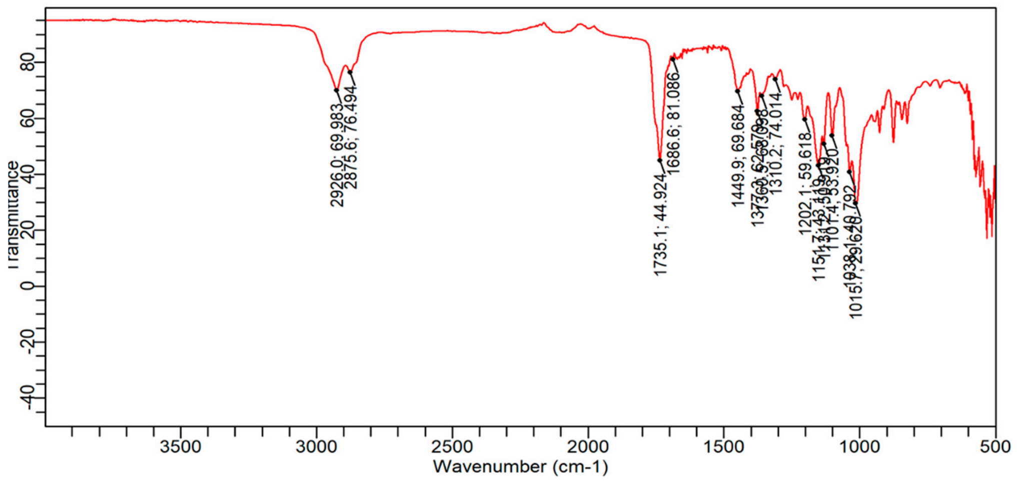

Transmittance
80
60
40
20
0
-20
-40
3500
3000
2500
2000
1500
1000
500
Wavenumber (cm-1)
2926.0; 69.983
2875.6; 76.494
1735.1; 44.924
1686.6; 81.086
1449.9; 69.684
1310.2; 74.014
1202.1; 59.618
1101.4; 53.920

*Hybrid 10b*

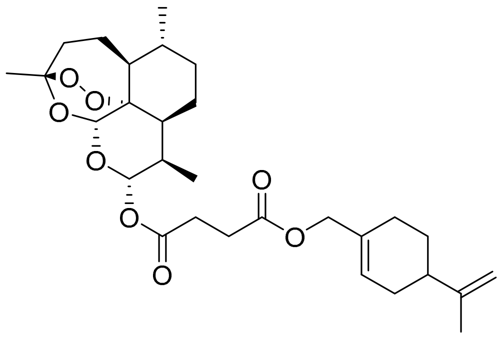

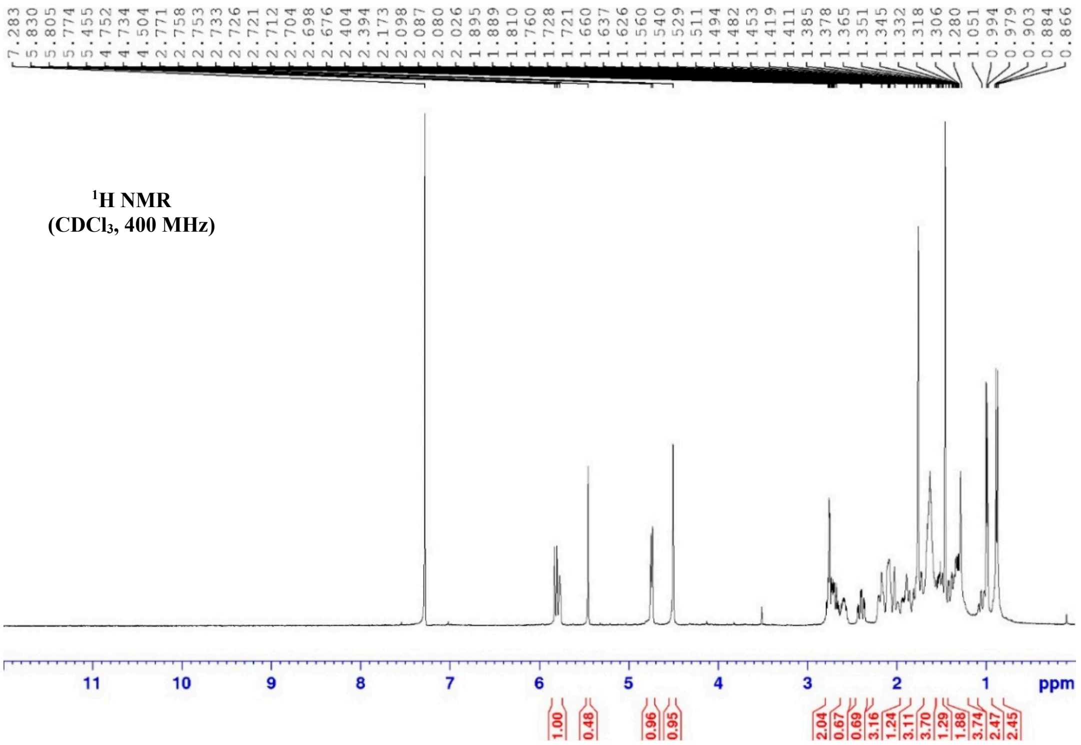
7.283 5.830 5.805 5.774 5.455 4.752 4.734 4.504 2.771 2.758 2.753 2.733 2.726 2.721 2.712 2.704 2.698 2.676 2.404 2.394 2.173 2.098 2.087 2.080 2.026 1.895 1.889 1.810 1.760 1.728 1.721 1.660 1.637 1.626 1.560 1.540 1.529 1.511 1.494 1.482 1.453 1.419 1.411 1.385 1.378 1.365 1.351 1.345 1.332 1.318 1.306 1.280 1.051 0.994 0.979 0.903 0.884 0.866
$^{1}$H NMR
($CDCl_3$, 400 MHz)
1.00 0.48 0.96 0.95 2.04 0.67 0.69 3.16 1.24 3.11 3.70 1.29 1.88 3.74 2.47 2.45
ppm

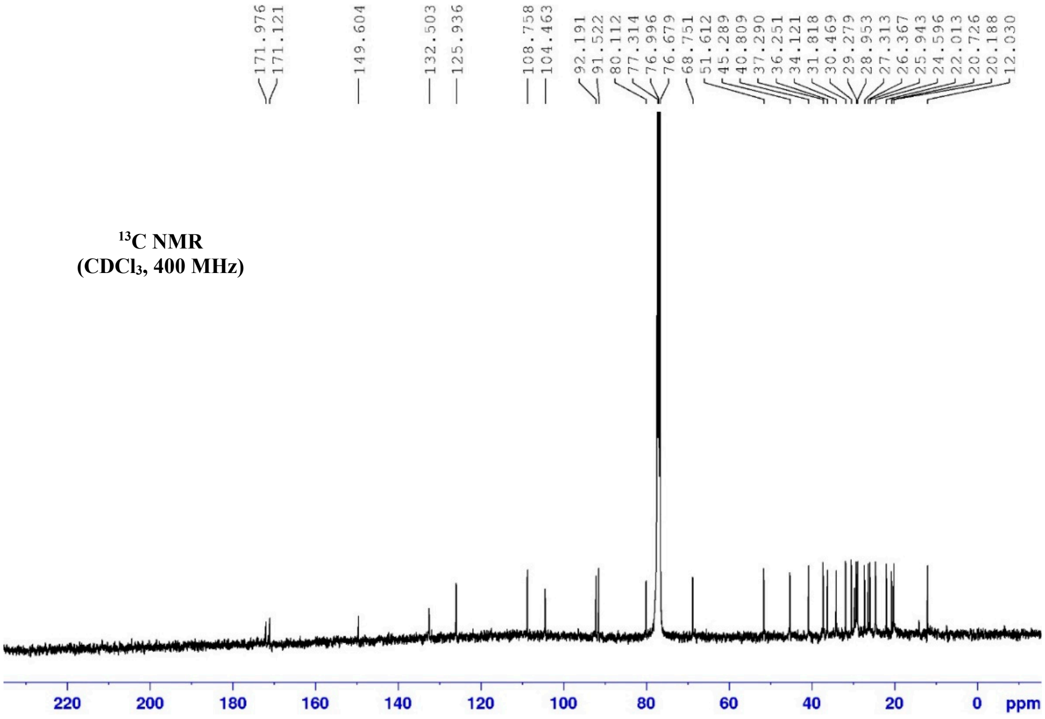
171.976 171.121 149.604 132.503 125.936 108.758 104.463 92.191 91.522 80.112 77.314 76.996 76.679 68.751 51.612 45.289 40.809 37.290 36.251 34.121 31.818 30.469 29.279 28.953 27.313 26.367 25.943 24.596 22.013 20.726 20.188 12.030
$^{13}$C NMR
($CDCl_3$, 400 MHz)
ppm

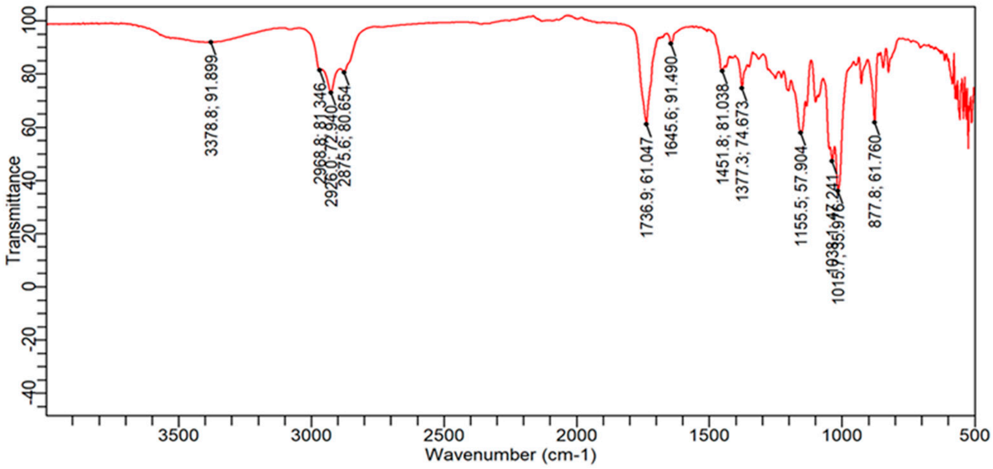
Transmittance
Wavenumber (cm-1)
3378.8; 91.899
2968.8; 81.346
2926.0; 72.940
2875.6; 80.654
1736.9; 61.047
1645.6; 91.490
1451.8; 81.038
1377.3; 74.673
1155.5; 57.904
1015.7; 35.976
877.8; 61.760

*Hybrid 10c*

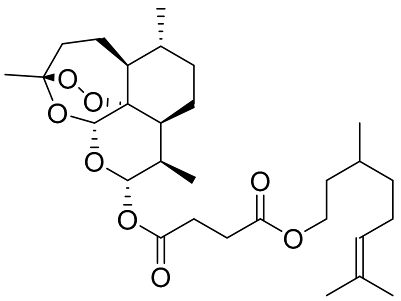

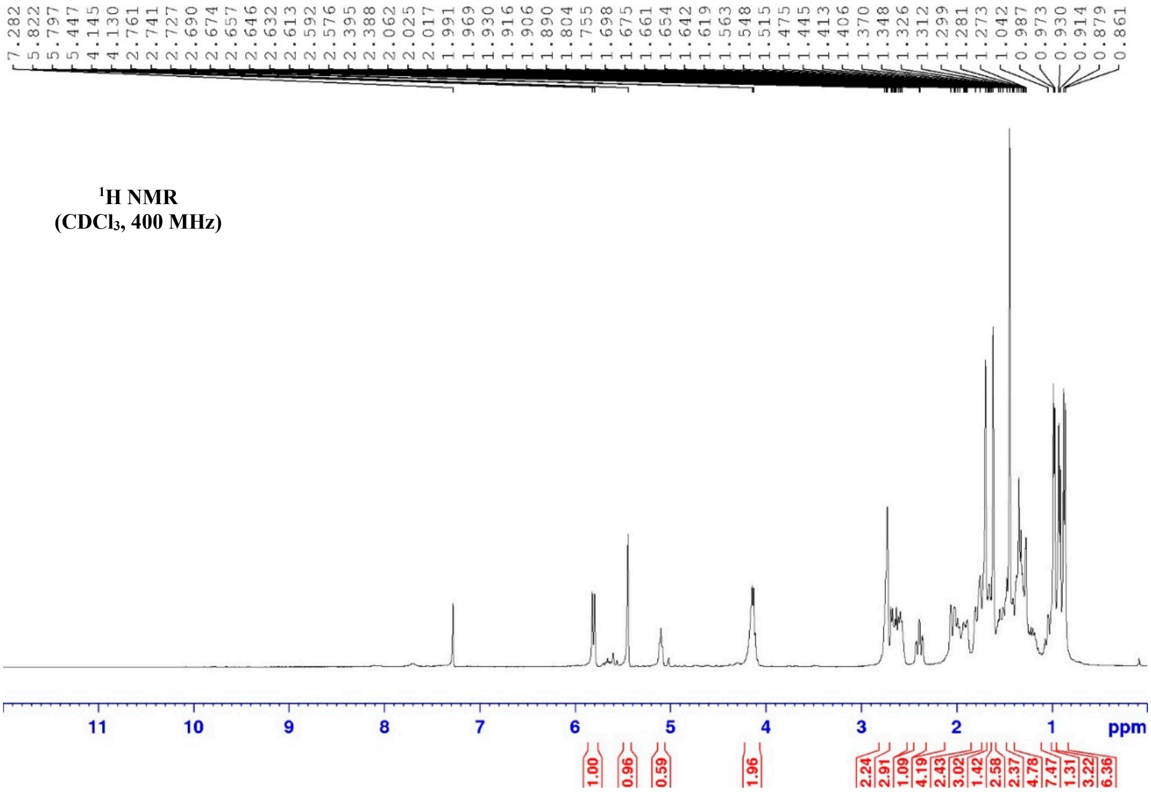


**$^{1}$H NMR**
**($CDCl_3$, 400 MHz)**

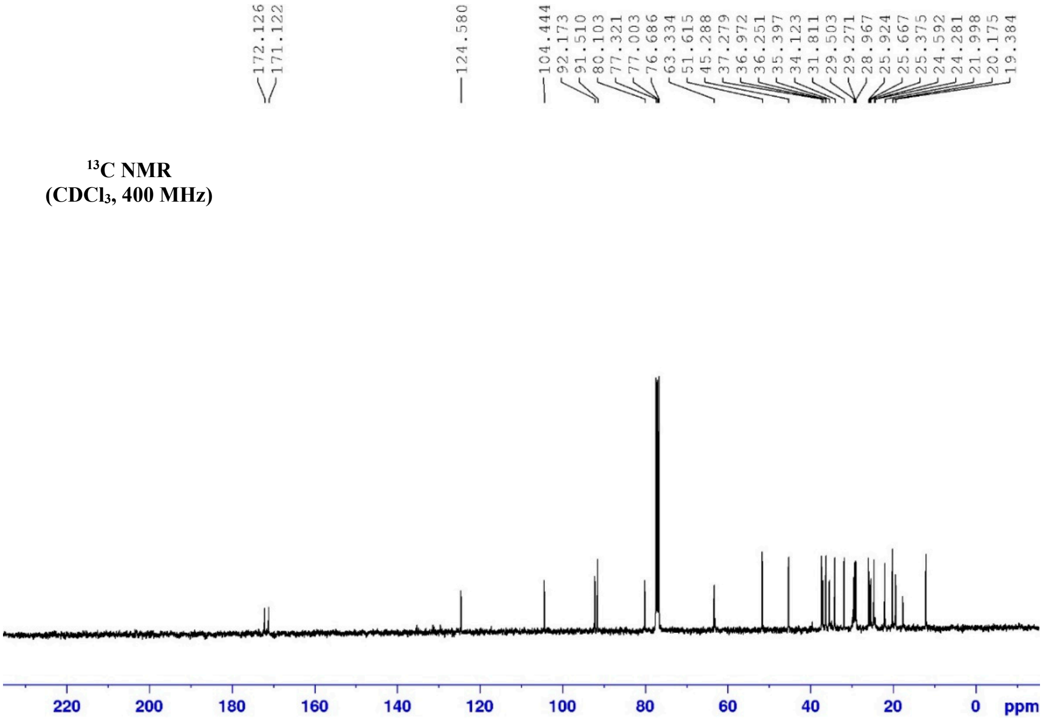


**$^{13}$C NMR**
**($CDCl_3$, 400 MHz)**

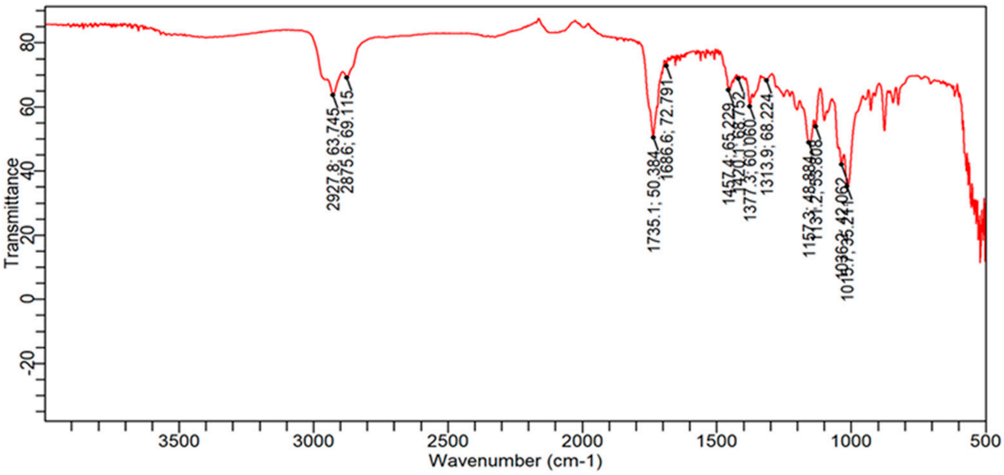

Transmittance
Wavenumber (cm-1)
2927.8; 63.745
2875.6; 69.115
1735.1; 50.384
1686.6; 72.791
1457.4; 65.229
1377.3; 60.060
1313.9; 68.224
1157.3; 48.884
1036.2; 42.062

***Hybrid 10d***

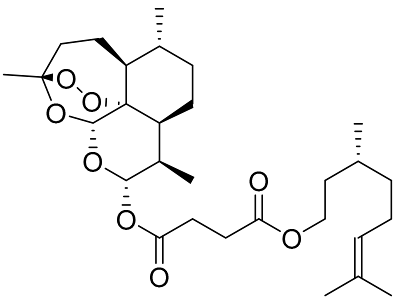

O
O
O
O
O
O
O
O


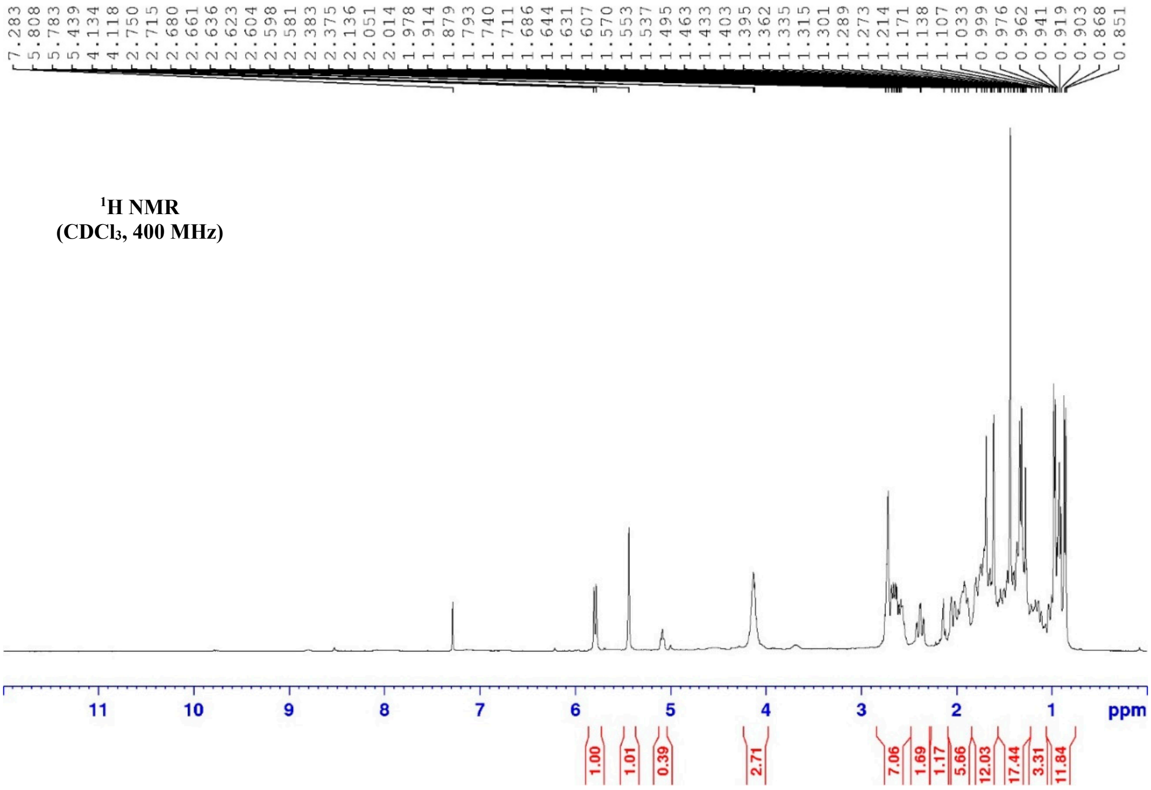

7.283
5.808
5.783
5.439
4.134
4.118
2.750
2.715
2.680
2.661
2.636
2.623
2.604
2.598
2.581
2.383
2.375
2.136
2.051
2.014
1.978
1.914
1.879
1.793
1.740
1.711
1.686
1.644
1.631
1.607
1.570
1.553
1.537
1.495
1.463
1.433
1.403
1.395
1.362
1.335
1.315
1.301
1.289
1.273
1.214
1.171
1.138
1.107
1.033
0.999
0.976
0.962
0.941
0.919
0.903
0.868
0.851
¹H NMR
(CDCl₃, 400 MHz)
11
10
9
8
7
6
5
4
3
2
1
ppm
1.00
1.01
0.39
2.71
7.06
1.69
1.17
5.66
12.03
17.44
3.31
11.84


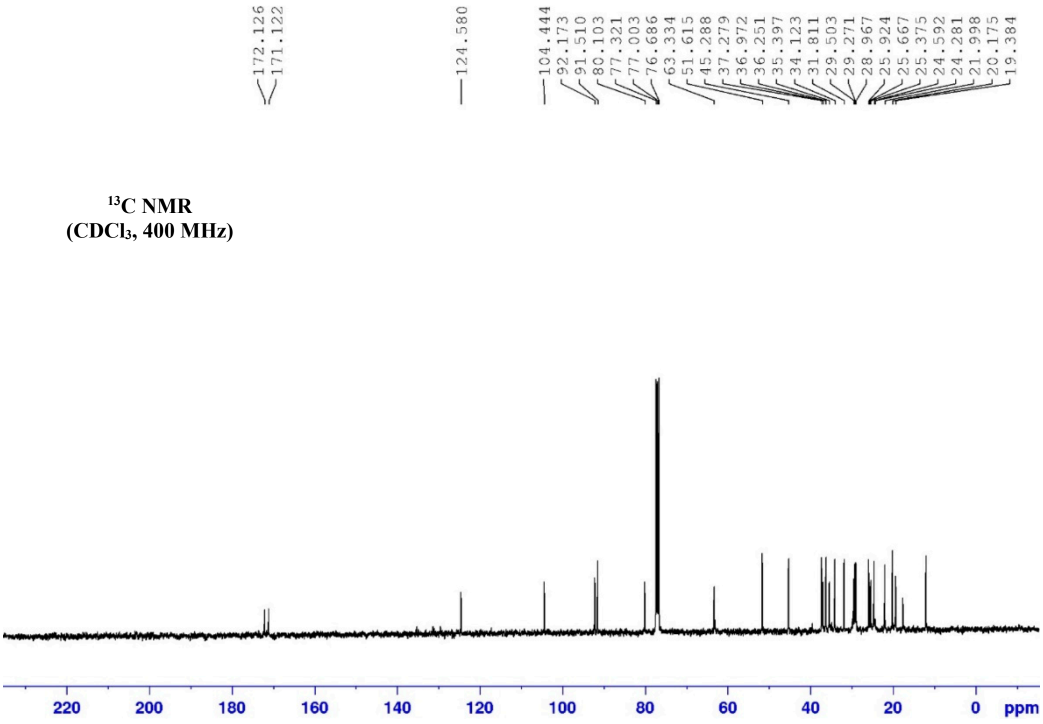

172.126
171.122
124.580
104.444
92.173
91.510
80.103
77.321
77.003
76.686
63.334
51.615
45.288
37.279
36.972
36.251
35.397
34.123
31.811
29.503
29.271
28.967
25.924
25.667
25.375
24.592
24.281
21.998
20.175
19.384
¹³C NMR
(CDCl₃, 400 MHz)
220
200
180
160
140
120
100
80
60
40
20
0
ppm

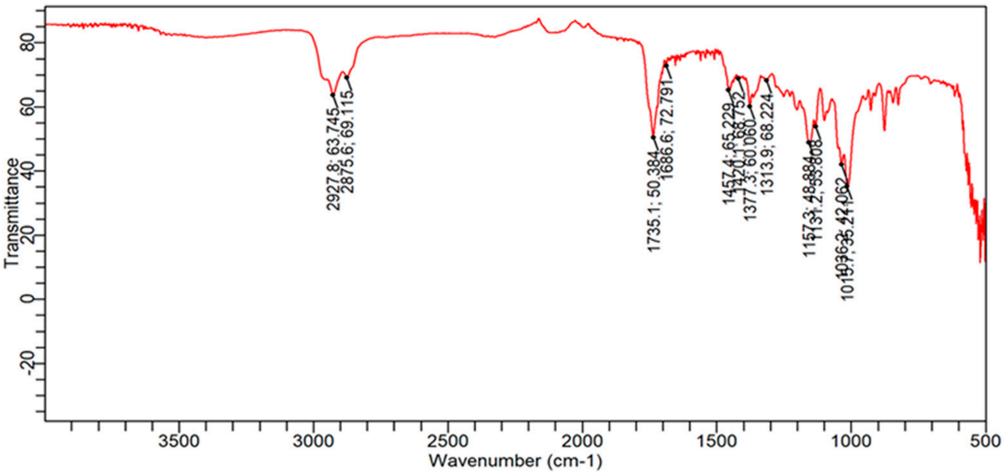

Transmittance
Wavenumber (cm-1)
80
60
40
20
0
-20
3500
3000
2500
2000
1500
1000
500
2927.8; 63.745
2875.6; 69.115
1735.1; 50.384
1686.6; 72.791
1457.4; 65.229
1377.3; 60.060
1313.9; 68.224
1157.3; 48.884

***Hybrid 10e***

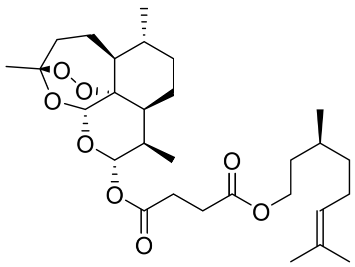

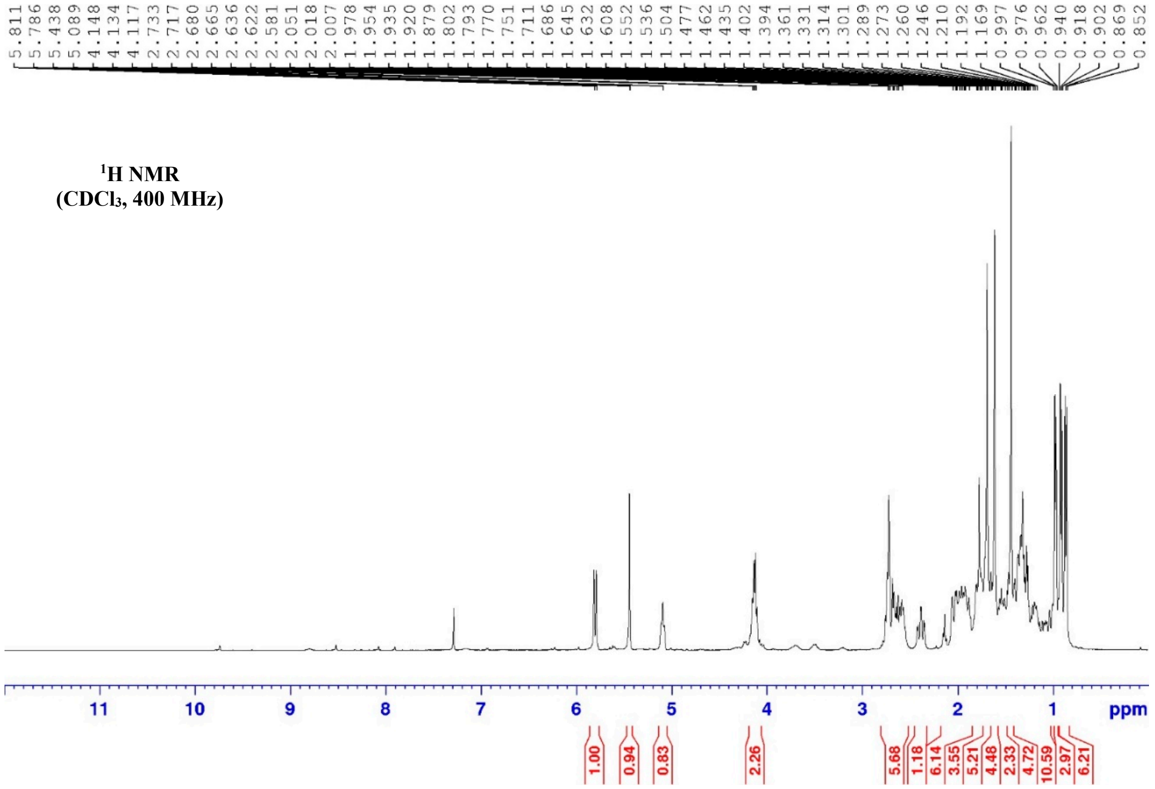
5.811
5.786
5.438
5.089
4.148
4.134
4.117
2.733
2.717
2.680
2.665
2.636
2.622
2.581
2.051
2.018
2.007
1.978
1.954
1.935
1.920
1.879
1.802
1.793
1.770
1.751
1.711
1.686
1.645
1.632
1.608
1.552
1.536
1.504
1.477
1.462
1.435
1.402
1.394
1.361
1.331
1.314
1.301
1.289
1.273
1.260
1.246
1.210
1.192
1.169
0.997
0.976
0.962
0.940
0.918
0.902
0.869
0.852
¹H NMR
(CDCl3, 400 MHz)
11
10
9
8
7
6
5
4
3
2
1
ppm
1.00
0.94
0.83
2.26
5.68
1.18
6.14
3.55
5.21
4.48
2.33
4.72
10.59
2.97
6.21

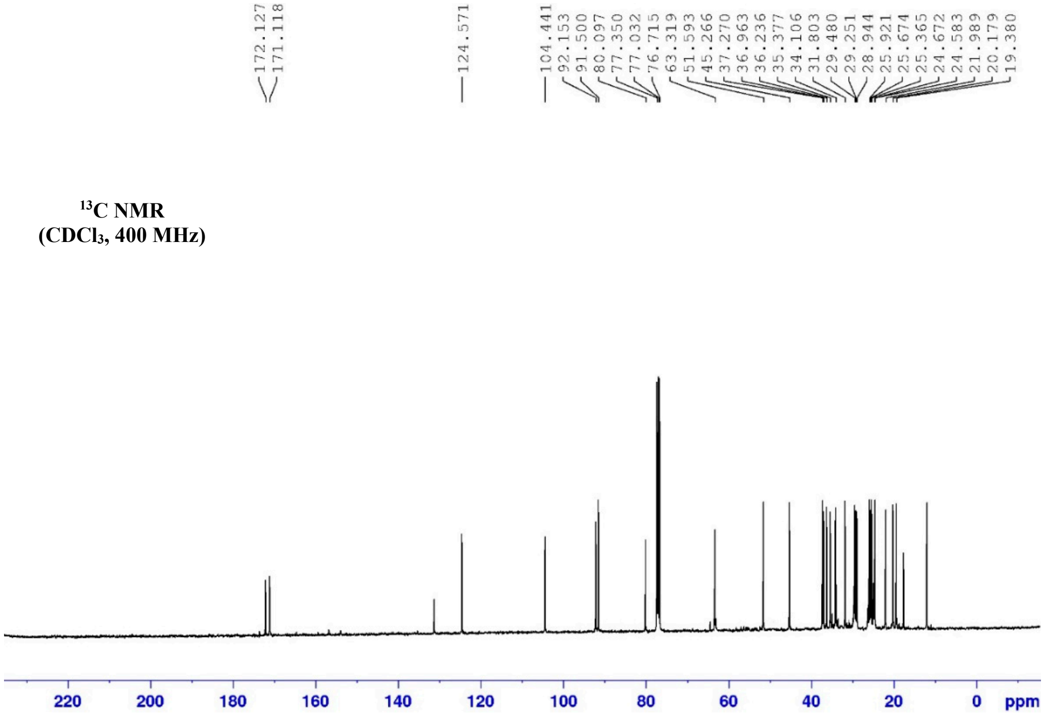
172.127
171.118
124.571
104.441
92.153
91.500
80.097
77.350
77.032
76.715
63.319
51.593
45.266
37.270
36.963
36.236
35.377
34.106
31.803
29.480
29.251
28.944
25.921
25.674
25.365
24.672
24.583
21.989
20.179
19.380
¹³C NMR
(CDCl3, 400 MHz)
220
200
180
160
140
120
100
80
60
40
20
0
ppm

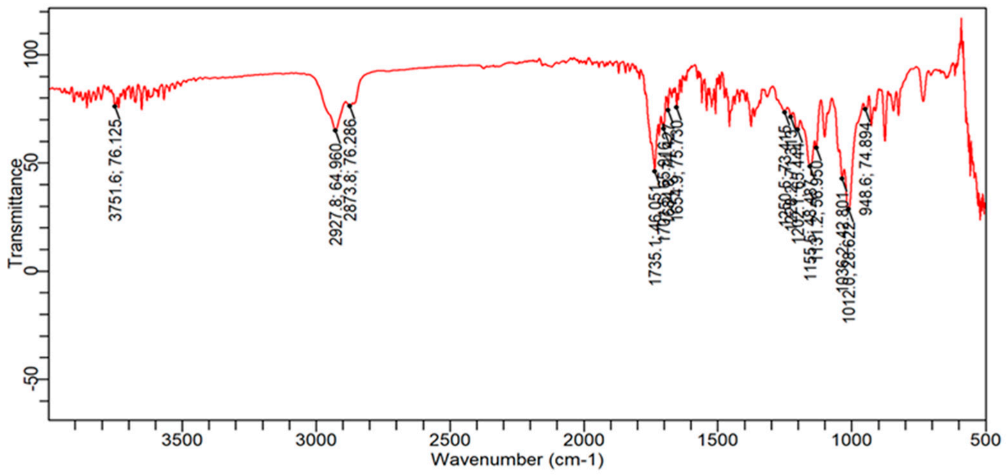

Transmittance
Wavenumber (cm-1)
100
50
0
-50
3500
3000
2500
2000
1500
1000
500
3751.6; 76.125
2927.8; 64.960
2873.8; 76.286
1735.1; 46.051
1654.9; 75.730
1131.2; 58.950
1012.0; 28.622
948.6; 74.894

*Hybrid 10f*

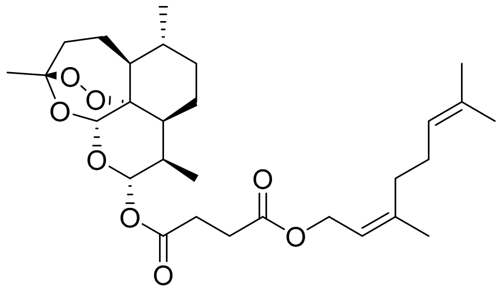

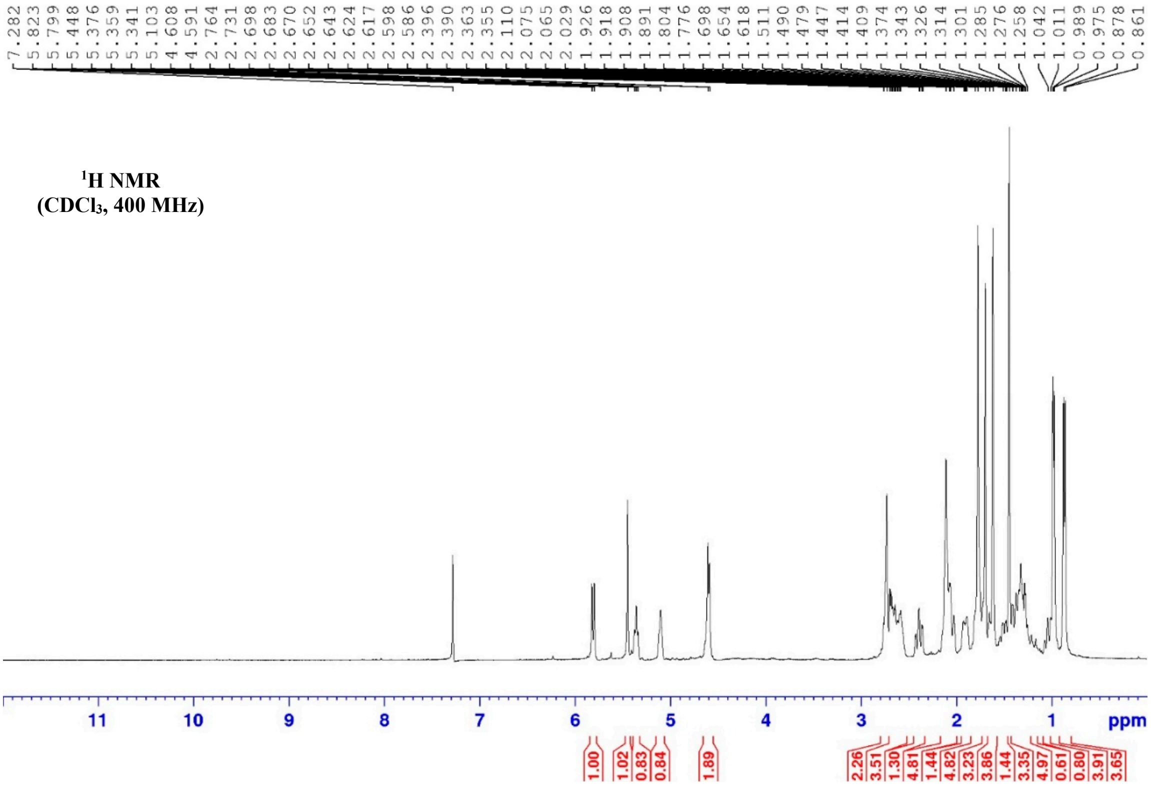
7.282
5.823
5.799
5.448
5.376
5.359
5.341
5.103
4.608
4.591
2.764
2.731
2.698
2.683
2.670
2.652
2.643
2.624
2.617
2.598
2.586
2.396
2.390
2.363
2.355
2.110
2.075
2.065
2.029
1.926
1.918
1.908
1.891
1.804
1.776
1.698
1.654
1.618
1.511
1.490
1.479
1.447
1.414
1.409
1.374
1.343
1.326
1.314
1.301
1.285
1.276
1.258
1.042
1.011
0.989
0.975
0.878
0.861
$^1$H NMR
(CDCl$_3$, 400 MHz)
11 10 9 8 7 6 5 4 3 2 1 ppm
1.00 1.02 0.83 0.84 1.89 2.26 3.51 1.30 4.81 1.44 4.82 3.23 3.86 1.44 3.35 4.97 0.61 0.80 3.91 3.65

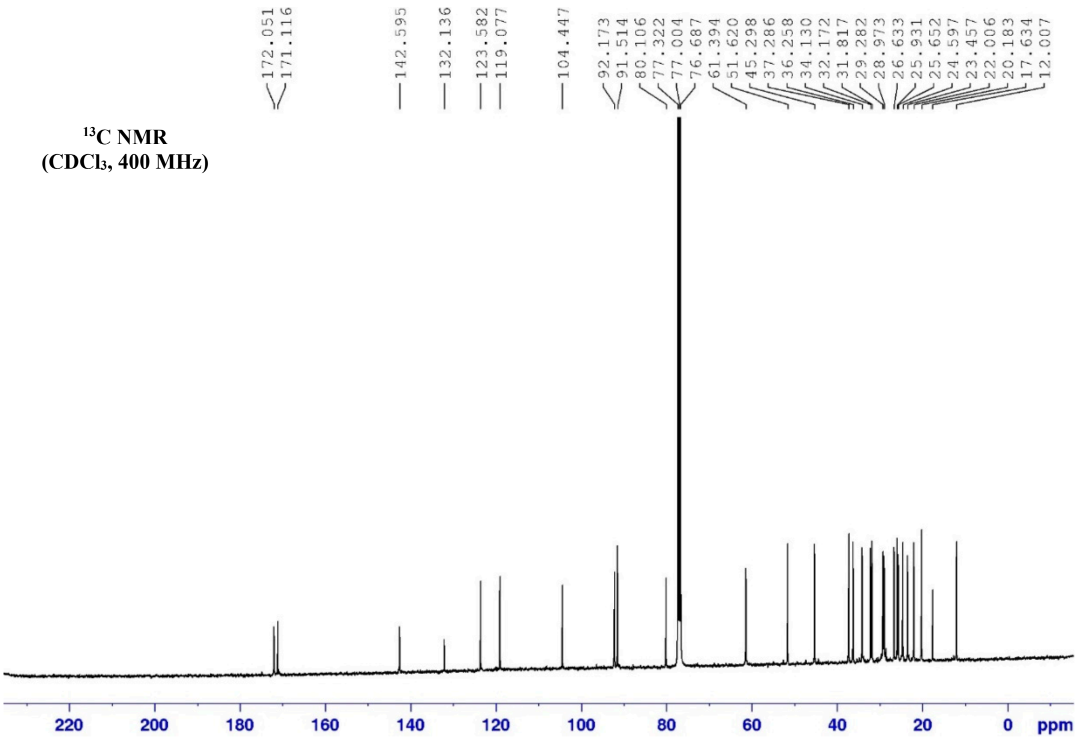
172.051
171.116
142.595
132.136
123.582
119.077
104.447
92.173
91.514
80.106
77.322
77.004
76.687
61.394
51.620
45.298
37.286
36.258
34.130
32.172
31.817
29.282
28.973
26.633
25.931
25.652
24.597
23.457
22.006
20.183
17.634
12.007
$^{13}$C NMR
(CDCl$_3$, 400 MHz)
220 200 180 160 140 120 100 80 60 40 20 0 ppm

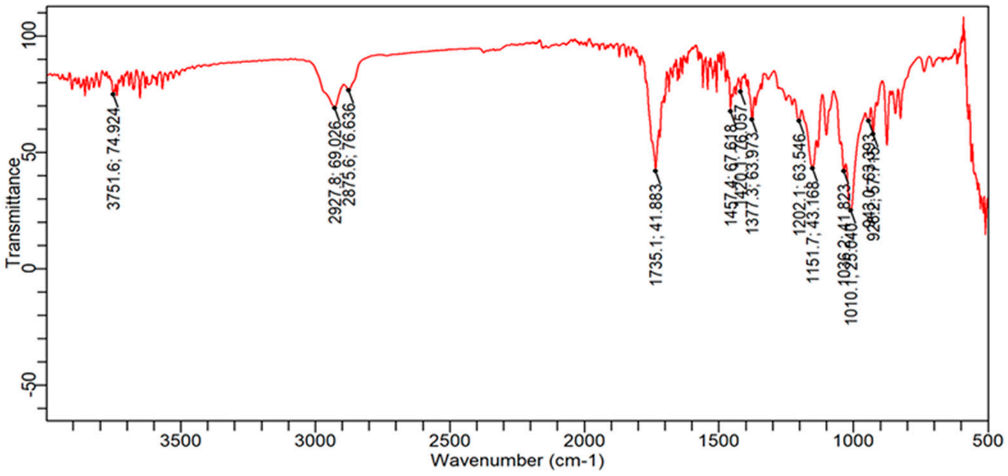
Transmittance
Wavenumber (cm-1)
100
50
0
-50
3500
3000
2500
2000
1500
1000
500
3751.6; 74.924
2927.8; 69.026
2875.6; 76.636
1735.1; 41.883
1377.3; 63.973
1202.1; 63.546
1151.7; 43.168
1010.1; 25.640

***Hybrid 10g***

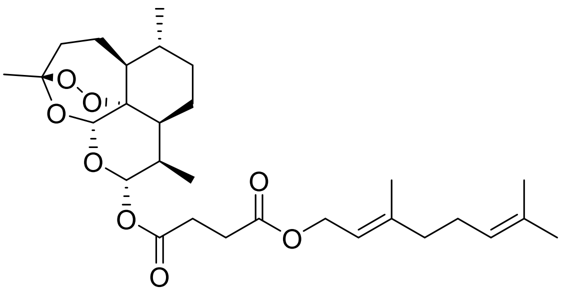

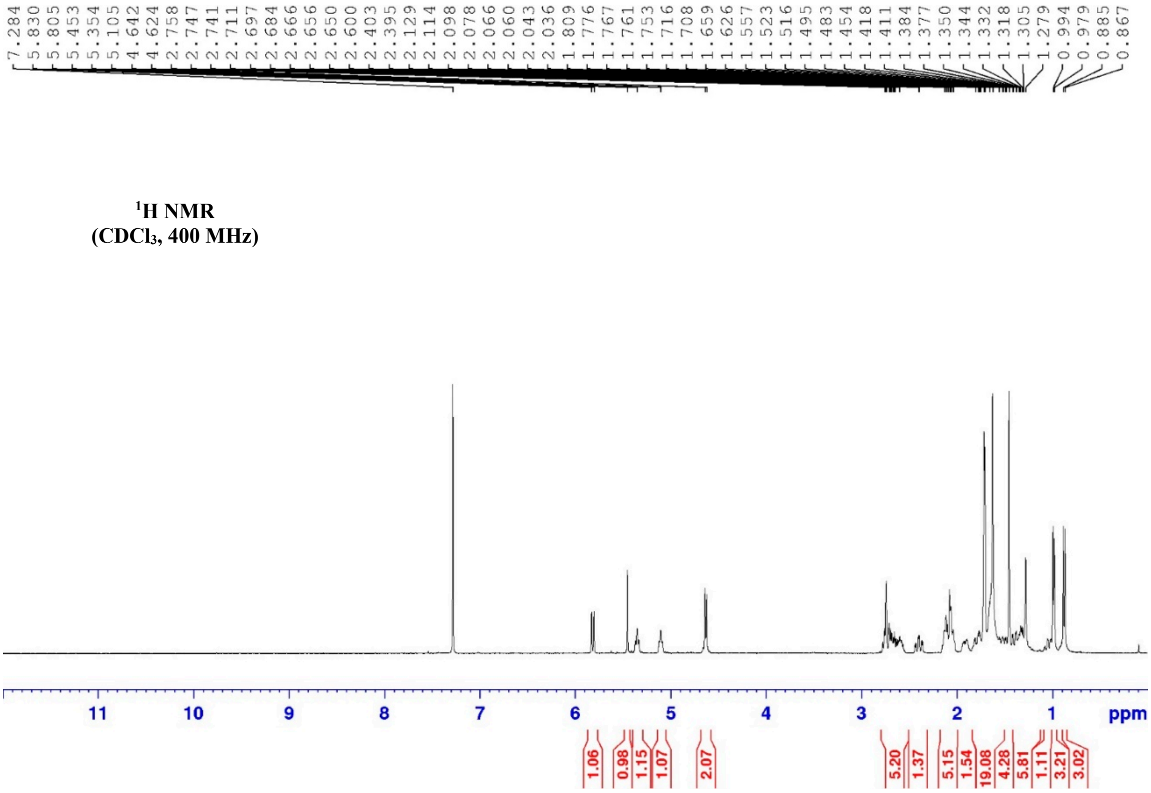


$^{1}$H NMR
(CDCl$_3$, 400 MHz)

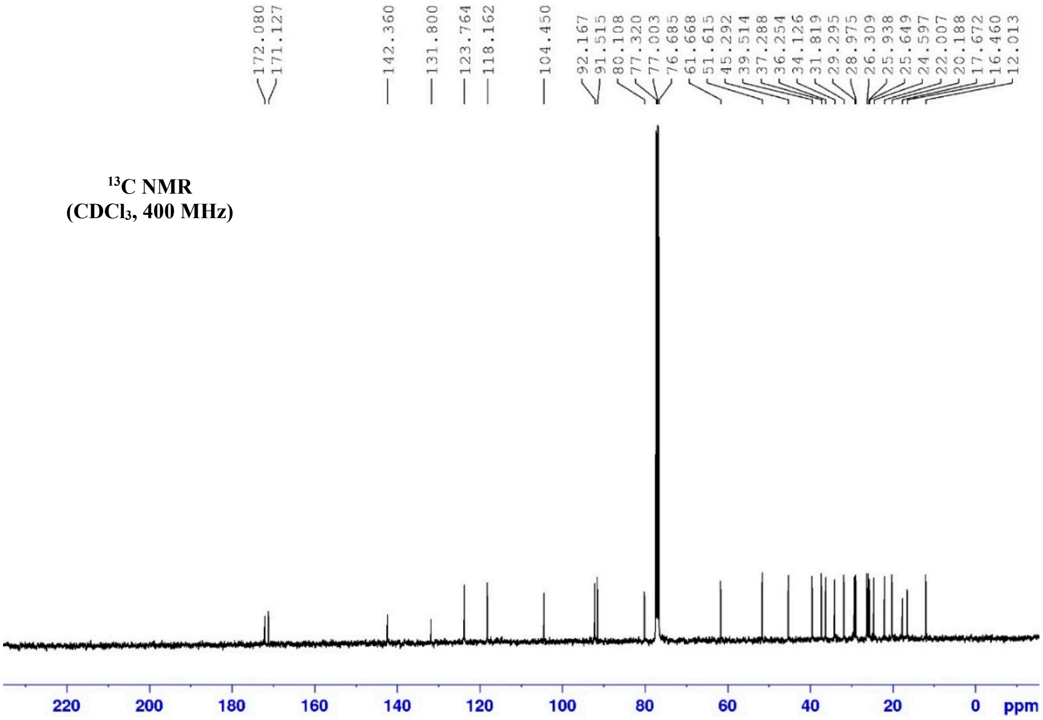


$^{13}$C NMR
(CDCl$_3$, 400 MHz)

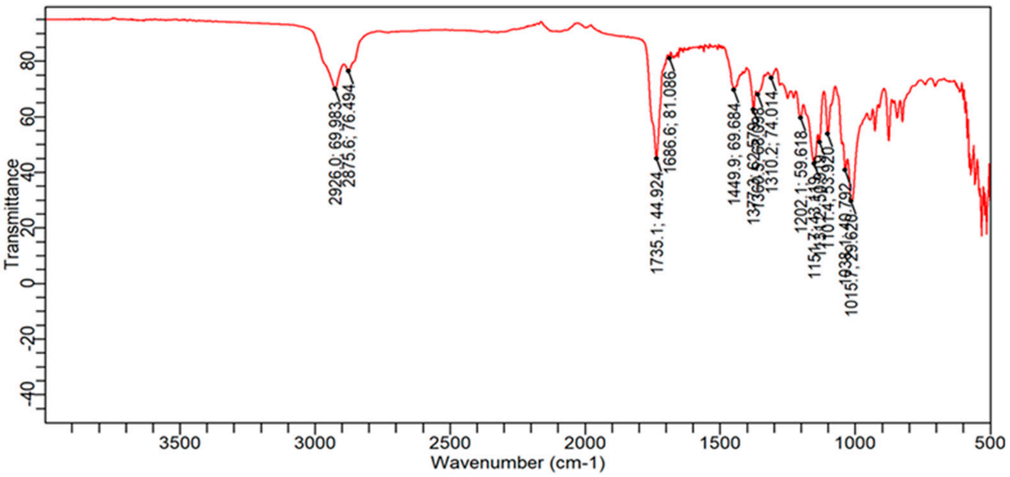

Transmittance
Wavenumber (cm-1)
80
60
40
20
0
-20
-40
3500
3000
2500
2000
1500
1000
500
2926.0; 69.983
2875.6; 76.494
1735.1; 44.924
1686.6; 81.086
1449.9; 69.684
1310.2; 74.014
1202.1; 59.618

## *2-deoxy-artemisinin derivatives 11c*

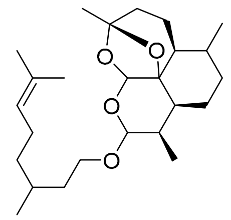

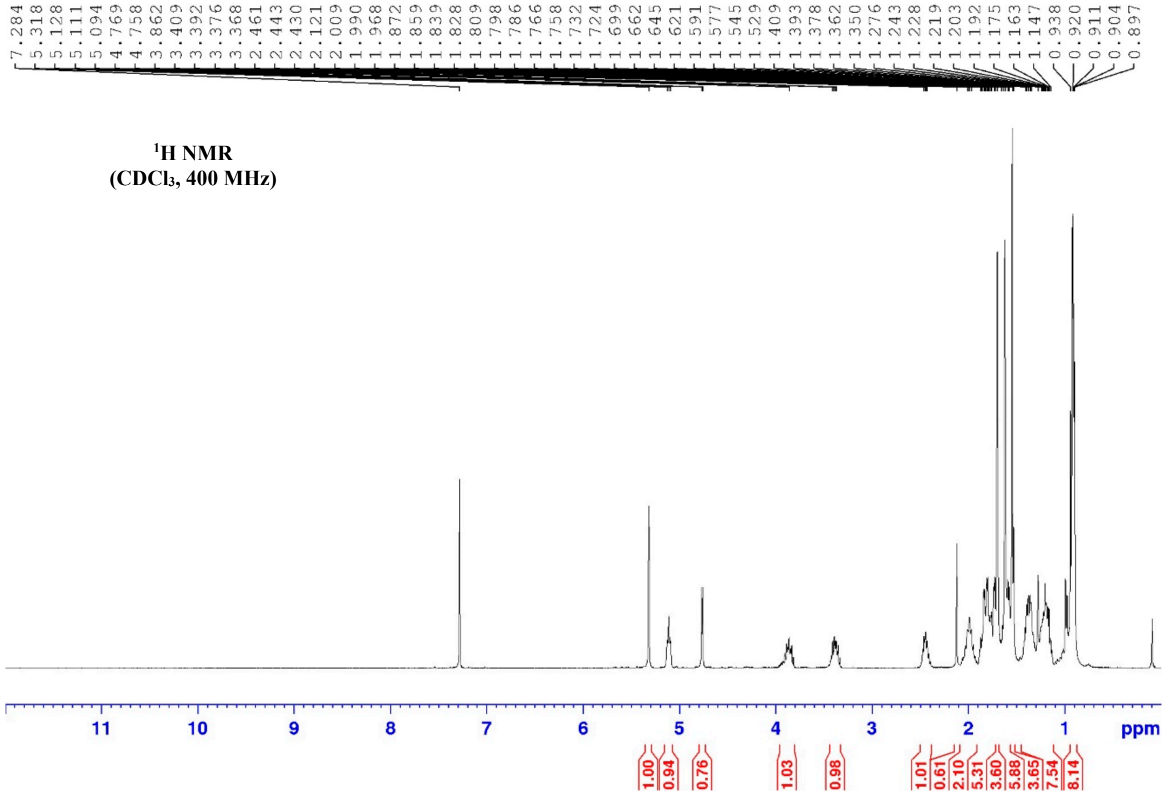


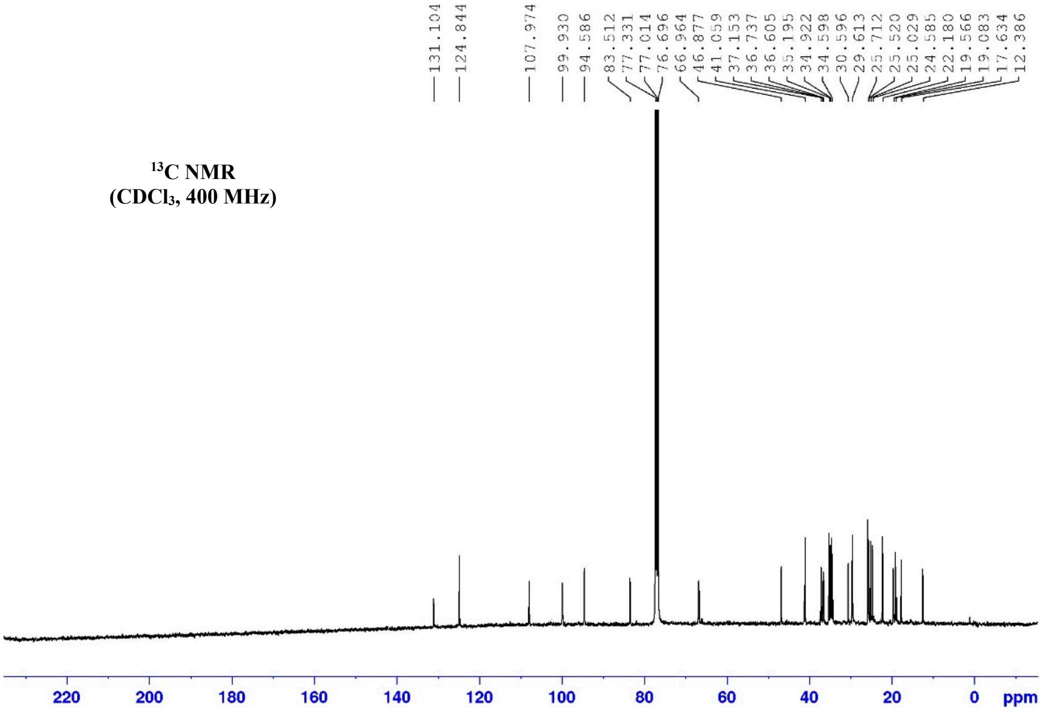

*2-deoxy-artemisinin derivatives 11f*

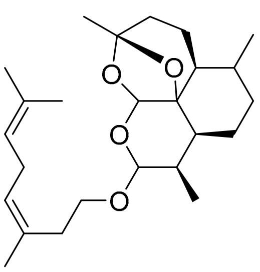

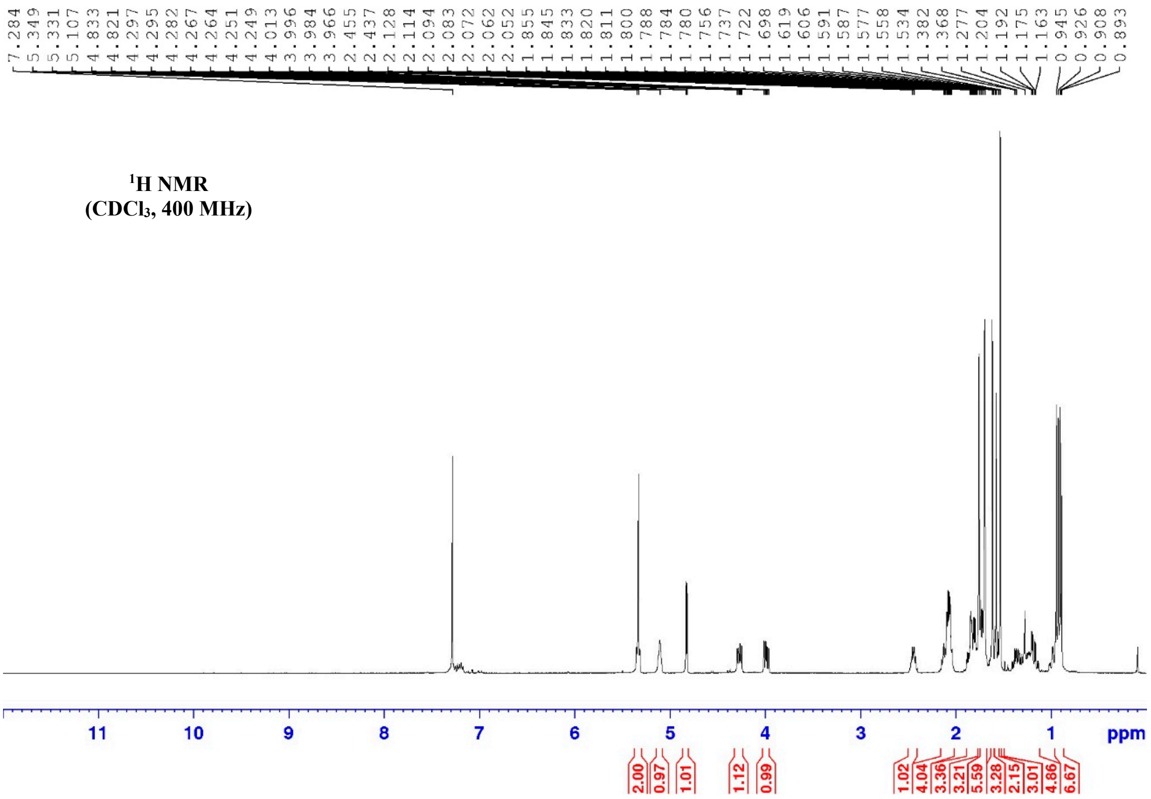


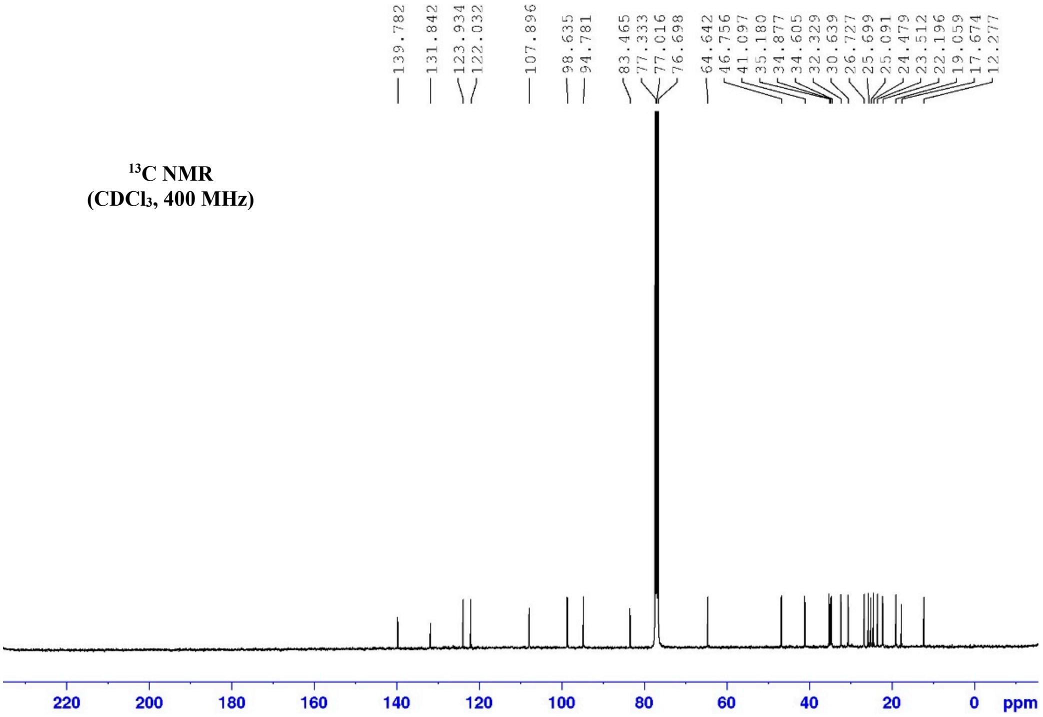

Supplement: Supplementary file 1 [file molecules-29-03421-s001.zip › molecules-3121446-supplementary.pdf]
